# PARP7-mediated ADP-ribosylation of FRA1 promotes cancer cell growth by repressing IRF1- and IRF3-dependent apoptosis

**DOI:** 10.1073/pnas.2309047120

**Published:** 2023-11-27

**Authors:** Patrick Manetsch, Flurina Böhi, Kathrin Nowak, Deena M. Leslie Pedrioli, Michael O. Hottiger

**Affiliations:** ^a^Department of Molecular Mechanisms of Disease, University of Zurich, 8057 Zurich, Switzerland; ^b^Molecular Life Science Ph.D. Program, Life Science Zurich Graduate School, University of Zurich, 8057 Zurich, Switzerland; ^c^Cancer Biology Ph.D. Program, Life Science Zurich Graduate School, University of Zurich, 8057 Zurich, Switzerland

**Keywords:** ADP-ribosylation, cancer, FRA1, proteasomal protein degradation, PARP7

## Abstract

PARP7 was reported to promote tumor growth in a cell-autonomous manner and by repressing the antitumor immune response. Nevertheless, the molecular mechanism of how PARP7-mediated ADP-ribosylation exerts these effects in cancer cells remains elusive. Here, we identified PARP7 as a nuclear and cysteine-specific mono-ADP-ribosyltransferase that modifies targets critical for regulating transcription, including the AP-1 transcription factor FRA1. Loss of FRA1 ADP-ribosylation via PARP7 inhibition by RBN-2397 or mutation of the ADP-ribosylation site C97 increased FRA1 degradation by the proteasome via PSMC3. The reduction in FRA1 protein levels promoted IRF1- and IRF3-dependent cytokine as well as proapoptotic gene expression, culminating in CASP8-mediated apoptosis. Furthermore, high PARP7 expression was indicative of the PARP7 inhibitor response in FRA1-positive lung and breast cancer cells. Collectively, our findings highlight the connected roles of PARP7 and FRA1 and emphasize the clinical potential of PARP7 inhibitors for FRA1-driven cancers.

## Significance

PARP7 inhibition has emerged as a compelling and novel option for cancer therapy in different tumor types. However, the molecular mechanism of how PARP7 inhibitors exert their antitumor effect has not yet been clarified. Here, we report that the transcription factor FRA1 is stabilized by PARP7-mediated ADP-ribosylation, thereby preventing its PSMC3-dependent proteasomal degradation and maintaining the suppression of IRF1- and IRF3-dependent gene expression. PARP7 inhibition consequently destabilizes FRA1 and allows for the expression of inflammatory and proapoptotic genes, culminating in CASP8-mediated apoptosis of cancer cells. This mechanism was verified with multiple lung and breast cancer cell lines, and the study demonstrated that in FRA1-driven cancer cells, PARP7 expression alone is necessary and sufficient to predict PARP7 inhibitor sensitivity.

ADP-ribosyltransferases (ARTs) are important regulators of the cellular immune response (1–3). The diphtheria toxin-like ARTs (also known as ARTD subfamily) comprise an enzyme family of 17 members and catalyze the transfer of ADP-ribose moieties from nicotinamide adenine dinucleotide (NAD^+^) to amino acids on target proteins (mono-ADP-ribosylation). Some ARTDs can extend the modification by adding further ADP-ribose moieties (poly-ADP-ribosylation) (4). Among the ARTDs, PARP7 (also known as TiPARP) has emerged as a critical repressor of the intratumoral immune response (5, 6). Initially, *PARP7* was reported to be the main target gene of the aryl hydrocarbon receptor (AHR) and to form a negative feedback loop by degrading AHR in an ADP-ribosylation-dependent manner (7–9). In addition, AHR-dependent expression of PARP7 was discovered to be important for constraining type I interferon (IFN) signaling in response to RNA viruses and nucleic acid (NA)-ligands (10). In cancer cells, genomic instability, a characteristic of almost all human cancers, is one of the main sources of cytoplasmic NA (11–13). The resulting innate immune response can restrain tumor growth; thus, cancer cells are under constant selective pressure to inhibit potentially deleterious NA-induced immune signaling (14). In this context, PARP7 inhibition by RBN-2397 restored cytoplasmic NA-dependent type I IFN signaling and reduced cancer cell growth in a cell-autonomous manner. RBN-2397 also contributed to tumor regression by enhancing cancer cell immune recognition in lung cancer xenografts and patients suffering from advanced solid tumors (5, 15).

At the molecular level, it was proposed that PARP7 exerted its repressive function on type I IFN signaling by ADP-ribosylation and inhibition of the TANK binding kinase 1 (TBK1) (10). However, a recent report highlighted that PARP7 regulates type I IFN signaling and tumor growth downstream of TBK1, thereby raising questions about the proposed mode of action of PARP7 inhibitors (6, 16). Moreover, the underlying cell death pathway(s) mediating the cell-autonomous effect of PARP7 inhibition on cancer cell survival were not yet defined. Thus, it remains crucial to identify PARP7 targets as potential biomarkers for patient stratification as well as to comprehensively understand how PARP7 inhibition affects tumor growth. Several strategies were recently developed to identify PARP7 substrates and gain insight into the molecular mechanism driving PARP7-dependency in cancer cells. However, rather than identifying the targets of endogenous PARP7, all reported approaches either identified PARP7 targets following the ectopic expression of PARP7 or using an engineered recombinant PARP7, thereby limiting the physiological relevance of the identified targets (17–19).

FRA1 *(FOSL1)* belongs to the AP-1 transcription factor family and is frequently overexpressed in tumors (20). The expression of FRA1 is critical for promoting cancer cell proliferation, growth, and invasion (21–23), and the FRA1 expression profile (i.e., FRA1-dependent genes) is a prognostic marker in multiple cancers (24). Moreover, constitutive mitogen-activated protein kinase (MAPK) signaling promotes the oncogenicity of FRA1 by inducing prolonged FRA1 expression and stabilizing FRA1 protein levels via C-terminal phosphorylation (24, 25). Intriguingly, the loss of FRA1 increases the expression of type I IFNs in breast cancer cells (26). Similarly, the downregulation of FRA1 in combination with poly(I:C) treatment further induces type I IFN expression, suggesting that FRA1 is a crucial transcriptional repressor of cytokine expression (26, 27). These findings indicate that controlling FRA1 expression may be a promising strategy for treating cancer. However, the pathways and, more importantly, the posttranslational modifications (PTM) governing FRA1 protein stability are not fully understood (25).

## Results

### PARP7 Localizes to the Nucleus and Modifies Transcriptional Regulators on Cysteine Residues.

**Fig. 1.**
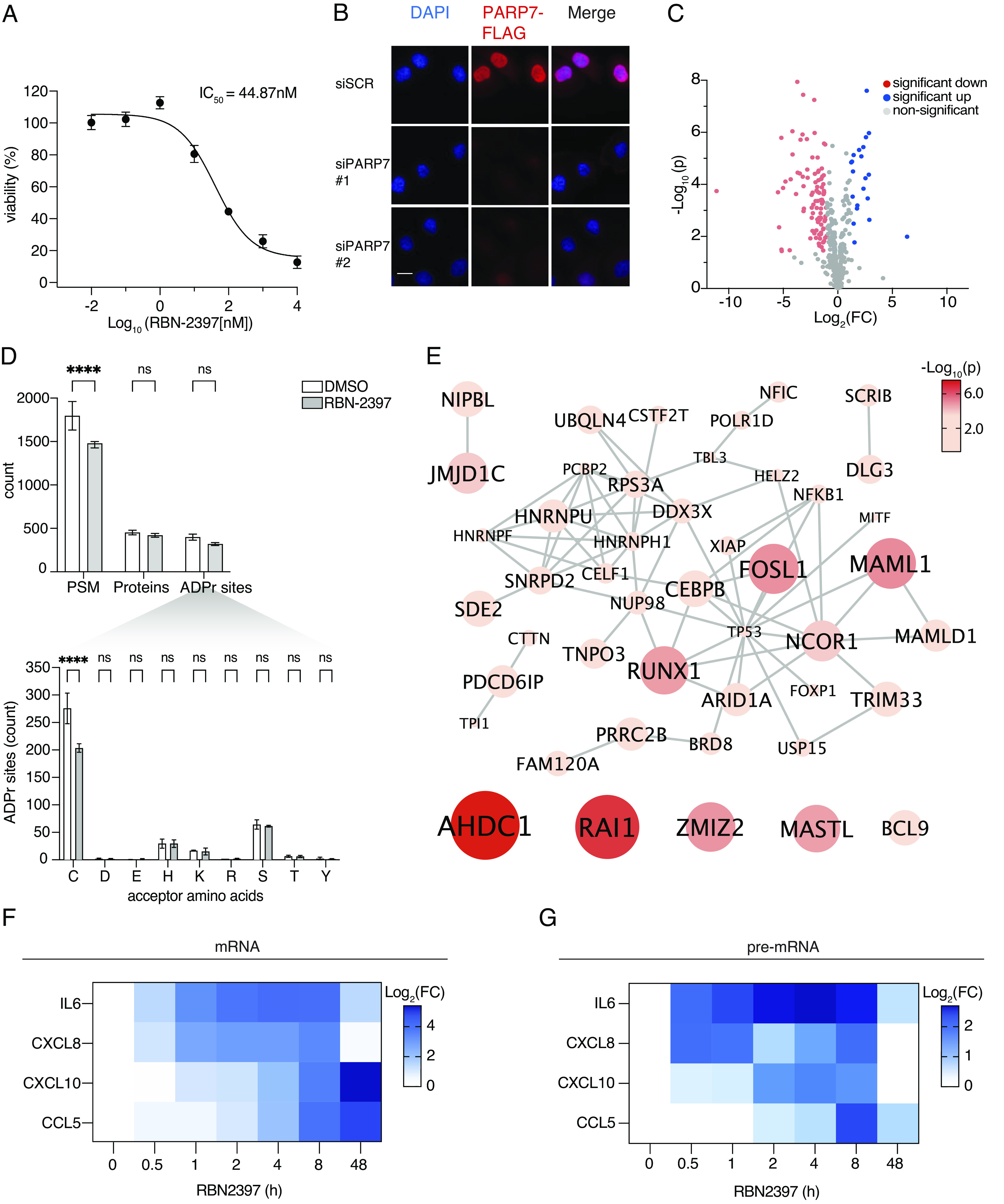
PARP7 controls transcription by ADP-ribosylation of its nuclear protein targets in NCI-H1975 cells. (*A*) Cell viability of NCI-H1975 cells was measured after six days of treatment with increasing concentrations of RBN-2397. Data are depicted as the mean ± SD of *N* = 3 biological replicates. Curves were fitted using a four-parametric nonlinear model. (*B*) IF analysis of endogenous FLAG-tagged PARP7 after the knockdown of PARP7 in NCI-H1975 cells. Representative image from a single experiment of *N* = 3 biological replicates. The scale bar represents 20 μm. (*C*) Volcano plot shows changes in ADP-ribosylation in NCI-H1975 cells treated with 100 nM RBN-2397 or DMSO for 24 h, *N* = 4 technical replicates. Red: significant down; blue: significant up; gray: nonsignificant. Significant changes were defined by FDR < 0.05 and FC ≥ ±1. (*D*) Bar graphs showing the count of unique ADPr-PSMs, unique ADPr-proteins, and unique ADPr-sites with ≥95% site-localization confidence (*Upper*). ADPr amino acid residue distribution was assessed by EThcD and HCD fragmentation (*Lower*). Data are shown as mean ± SD of *N* = 4 technical replicates. (*E*) STRING network visualization of proteins exhibiting a significant decrease in ADP-ribosylation after RBN-2397 treatment in NCI-H1975 cells (Node size and color: −Log_10_(*P*)). Default STRING clustering confidence was used (*P* > 0.4), and disconnected proteins were omitted from the network unless they were identified by FDR < 0.05 and FC ≥ 2. (*F* and *G*) Heat maps showing RT-qPCR analysis of mRNA and pre-mRNA levels in NCI-H1975 cells treated with RBN-2397 for the indicated periods. The data are represented as the mean Log_2_(FC) of *N* = 5 and *N* = 3 biological replicates, respectively.

Previous clinical trials have demonstrated that the PARP7 inhibitor RBN-2397 is well tolerated and displays preliminary antitumor activity in patients with advanced solid tumors (15). However, the endogenous targets of PARP7 and the molecular mechanism of PARP7 dependency remain unknown. To understand the function of PARP7 in cancer cells, we selected the lung adenocarcinoma cell line NCI-H1975, which was previously described as sensitive to PARP7 knockout (https://depmap.org/portal/). Indeed, PARP7 inhibition for six days and knockdown for three days strongly reduced the cell viability of NCI-H1975 cells, thereby confirming their PARP7 dependency (Fig. 1*A* and *SI Appendix*, Fig. S1 *A* and *B*). Since protein localization and function are tightly interconnected, we first aimed to determine the cellular localization of PARP7. Endogenous PARP7 predominantly localized to the nucleus as observed by confocal immunofluorescence (IF) analysis (Fig. 1*B*), and the nuclear localization of PARP7 and PARP7-mediated ADP-ribosylation was further confirmed by ectopically expressing HA-tagged PARP7 in A549 cells using a Doxycycline (Dox)-inducible construct (*SI Appendix*, Fig. S1*C*). Together, these results suggest that PARP7 and PARP7-mediated ADP-ribosylation predominantly localize to the nucleus.

To elucidate how PARP7-mediated ADP-ribosylation contributes to cell viability, we identified endogenous PARP7 target proteins using label-free quantification (LFQ) tandem mass spectrometry (LC–MS/MS) (28). In short, we treated NCI-H1975 cells with RBN-2397 or DMSO for 24 h, followed by the enrichment of ADP-ribosylated peptides for LC–MS/MS analyses. The quantification of ADP-ribosylated peptides revealed that PARP7 inhibition significantly decreased the modification of 85 unique proteins (Fig. 1*C* and Dataset S1). Surprisingly, RBN-2397 treatment also led to a significant increase in the modification of 19 unique proteins, including the ADP-ribosyltransferase PARP14, suggesting that PARP7 inhibits the activity of other ARTs (Fig. 1*C*). Considering that proteins exhibiting increased ADP-ribosylation after PARP7 inhibition are unlikely to be direct targets of PARP7, they were not further pursued here. Interestingly, RBN-2397 treatment significantly reduced the modification on cysteine residues and not on the other potential ADP-ribosylation acceptor sites analyzed (Fig. 1*D*). Consistent with these findings, we observed that overexpressed PARP7 in A549 cells led to the modification of proteins almost exclusively on cysteines, which was abrogated upon PARP7 inhibition by RBN-2397 (*SI Appendix*, Fig. S1*D* and Dataset S2).

To gain insight into the cellular functions of the PARP7 target proteins identified here, we performed a STRING network analysis of the PARP7 targets that exhibited a significant loss in ADP-ribosylation after RBN-2397 treatment of NCI-H1975 cells (Fig. 1*E*). We found that the majority of these PARP7 targets localize to the nucleus and are involved in the regulation of gene expression (*SI Appendix*, Fig. S1 *E* and *F*), corroborating the observed nuclear localization of PARP7 (Fig. 1*B*). In support of this finding, a significant enrichment of GO terms related to nuclear localization and the regulation of gene expression was observed in PARP7 overexpressing A549 cells (*SI Appendix*, Fig. S1 *G* and *H*). To confirm the regulatory role of PARP7 in gene expression, we analyzed transcriptional changes of four PARP7-dependent proinflammatory genes (5) at different time points after PARP7 inhibition in NCI-H1975 cells. RBN-2397 treatment resulted in a significant and immediate upregulation (Log_2_(FC) ≥ 2 after 1 h) of *IL6* and *CXCL8* (Fig. 1*F*). At the same time, *CXCL10* and *CCL5* were only up-regulated after prolonged (Log_2_(FC) ≥ 2 after 8 h) RBN-2397 treatment periods (Fig. 1*F*). The same time-dependent expression pattern was observed by analyzing the pre-mRNA levels of these genes (Fig. 1*G*), which indicates two distinct waves of gene expression rather than a difference in pre-mRNA stability. Together, these observations suggest that the upregulation of *IL6* and *CXCL8* is an immediate response to PARP7 inhibition. In contrast, the late upregulation of *CXCL10* and *CCL5* pre- and mRNA levels indicates that these genes are not directly transcriptionally regulated by PARP7 but are likely up-regulated through signaling events activated by PARP7 inhibition. In conclusion, these results provide evidence that PARP7 controls transcription both directly and indirectly through the ADP-ribosylation of its nuclear targets.

### The Cellular Sensitivity to PARP7 Inhibition Is Dependent on FRA1.

**Fig. 2.**
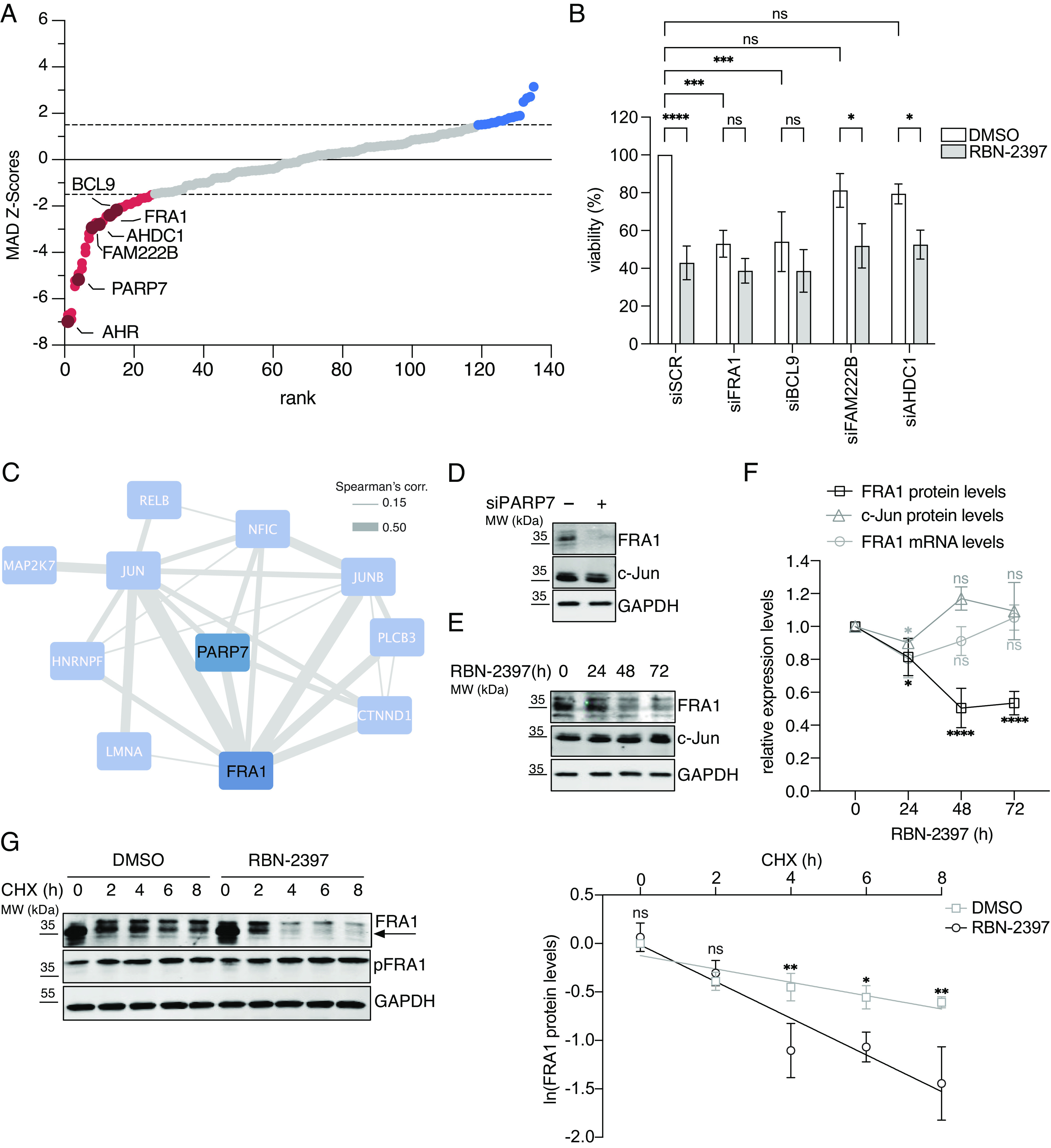
The PARP7 inhibition-dependent decline in cell viability is mediated by the degradation of FRA1. (*A*) Waterfall plot showing the robust z-transformed (MAD z-score) SI (sensitivity index) for the mean of each siRNA of *N* = 3 biological replicates. Red: <−1.5; blue >1.5; dark red: genes of which two out of three siRNA showed a robust z-score ≤ −1.5. (*B*) Cell viability of NCI-H1975 cells 5 d after siRNA transfection and RBN-2397 treatment. The data are normalized to the siSCR + DMSO control and shown as the mean ± SD of *N* = 3 biological replicates. (*C*) STRING network visualizes the genetic codependencies between genes that exhibited a strong correlation in the DepMap dataset (Spearman ≥ 0.1; edge size: strength of Spearman’s correlation). (*D*) Immunoblot of NCI-H1975 cells following 48 h PARP7 knockdown. Representative image of *N* = 3 biological replicates. (*E* and *F*) Immunoblot of NCI-H1975 cells treated with RBN-2397 for the indicated periods. Quantification of FRA1 and c-Jun immunoblots and FRA1 mRNA is shown as the mean ± SD of *N* = 3 biological replicates. (*G*) Immunoblot of NCI-H1975 cells treated first with RBN-2397 for 24 h and then with CHX (50 μg/mL) and RBN-2397 for the indicated periods (*Left*). Quantification of FRA1 immunoblots is shown as the mean ± SD of *N* = 3 biological replicates (*Right*).

To investigate which of the identified PARP7 targets are involved in the RBN-2397-mediated decrease in cell viability, we performed a siRNA screen to knockdown 45 identified PARP7 targets with the strongest reduction in ADP-ribosylation after RBN-2397 treatment. As expected, PARP7 knockdown resulted in reduced sensitivity to RBN-2397, suggesting that the decrease in cell viability observed in NCI-H1975 cells is a direct consequence of PARP7 inhibition (Fig. 2*A*). Likewise, the knockdown of AHR, a regulator of PARP7 expression (*SI Appendix*, Fig. S2*A*), reduced RBN-2397 sensitivity (Fig. 2*A*). Among the ADP-ribosylated targets of PARP7, knockdown of AHDC1, FAM222B, BCL9, and FRA1 strongly reduced RBN-2397 sensitivity in NCI-H1975 cells (Fig. 2*A*). To confirm that AHDC1, FAM222B, BCL9 and FRA1 reduce the cytotoxic effect of RBN-2397, we analyzed cell viability after siRNA-mediated knockdowns of all four candidate genes and following the treatment with RBN-2397 or DMSO (Fig. 2*B* and *SI Appendix*, Fig. S2*C*). As a positive control for cell death, we used a siRNA targeting the common essential gene *PLK1,* and as a control for the reduced cellular sensitivity to RBN-2397, we again knocked down AHR (*SI Appendix*, Fig. S2 *B* and *C*). Remarkably, we observed that only the knockdown of FRA1 and BCL9 reduced the cellular sensitivity to RBN-2397, while cells retained their sensitivity to PARP7 inhibition following the depletion of FAM222B and AHDC1 (Fig. 2*B* and *SI Appendix*, Fig. S2*C*). This finding suggests that FRA1 and/or BCL9 contribute to the RBN-2397-mediated decrease in cell viability. To further investigate whether FRA1 or BCL9 regulates cell viability downstream of PARP7 activity, we compared the genetic dependencies of PARP7 and its protein targets across different cell lines using ShinyDepMap (29). Genes are characterized as codependent if their effects on cell viability positively correlate. Interestingly, PARP7 clustered only with FRA1, which is ADP-ribosylated by PARP7 on C97, thus indicating that these two proteins regulate the same pathways (Fig. 2*C*). Since FRA1 knockdown alone decreased cell viability, but additional PARP7 inhibition did not further affect viability, PARP7 likely functions upstream of FRA1 and promotes cell survival through the ADP-ribosylation of FRA1 (Fig. 2*B*).

### PARP7 Inhibition Promotes the Degradation of FRA1.

FRA1 belongs to the AP-1 transcription factor family and is frequently overexpressed in tumors (20). Moreover, the oncogenicity of FRA1 is promoted by PTM-mediated stabilization (30). Interestingly, immunoblot and immunofluorescence analyses demonstrated that PARP7 knockdown and RBN-2397 treatment significantly reduced FRA1 protein levels (Fig. 2 *D*–*F* and *SI Appendix*, Fig. S2 *D*–*F*). In contrast, PARP7 knockdown or inhibition did not affect c-Jun protein levels, another AP-1 transcription factor with a similar genetic codependency as FRA1 (Fig. 2 *D*–*F* and *SI Appendix*, Fig. S2*D*). Moreover, while PARP7 inhibition decreased FRA1 protein levels, FRA1 mRNA levels were not reduced in the same period, suggesting that PARP7-mediated ADP-ribosylation specifically regulates FRA1 protein levels (Fig. 2*F*). To explore whether PARP7-mediated ADP-ribosylation directly stabilizes FRA1, we measured FRA1 degradation rates after DMSO or RBN-2397 pretreatment for 24 h using the translation inhibitor cycloheximide (CHX). RBN-2397 treatment significantly enhanced FRA1 degradation, suggesting that the enzymatic activity of PARP7 is required for FRA1 stabilization (Fig. 2*G*). Remarkably, we found that the lower-migrating isoform of FRA1 was preferentially degraded compared to the higher-migrating isoform, which corresponded to the phosphorylated FRA1 isoform (Fig. 2*G*). These findings suggest that PARP7 activity primarily stabilizes the unphosphorylated isoform of FRA1.

### ADP-Ribosylation of FRA1 Prevents Its PSMC3-Dependent Proteasomal Degradation.

**Fig. 3.**
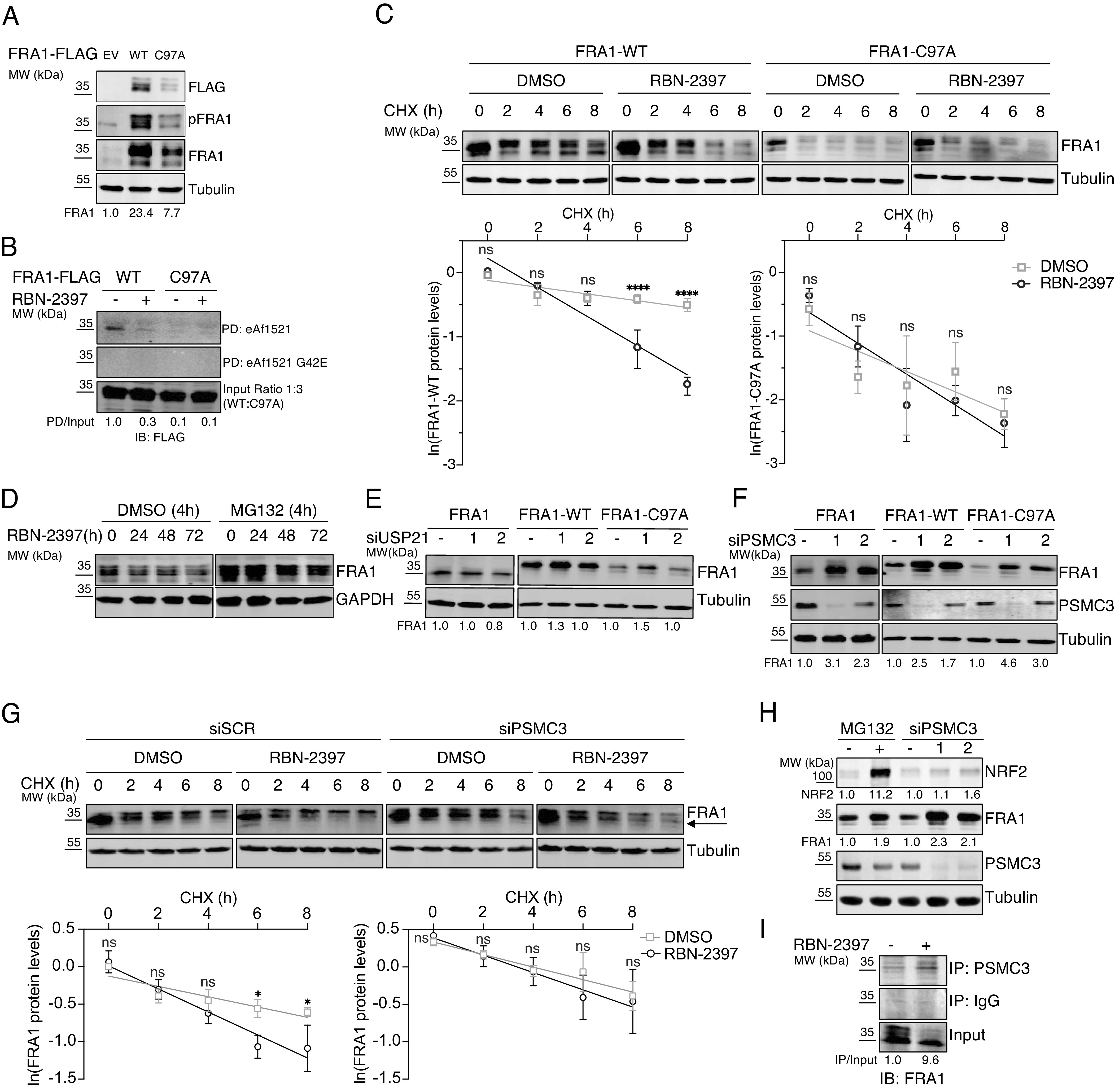
ADP-ribosylation of FRA1 on C97 reduces its degradation by PSMC3 and the proteasome. (*A*) Immunoblot of NCI-H1975 cells transduced with lentiviral constructs for empty vector (EV), FRA1-WT, or C97A and expressed under a constitutive promotor. Representative image from a single experiment with *N* = 3 biological replicates. Quantification of FRA1 immunoblots is indicated as the average of *N* = 3. (*B*) Immunoblot following eAf1521-dependent pull-down (PD) of ectopically expressed FRA1-WT or -C97A in NCI-H1975 cells. Representative image from a single experiment with *N* = 3 biological replicates. Quantification of FRA1 PD immunoblots was performed by normalizing to the FRA1 input and is indicated as the average of *N* = 3. (*C*) Immunoblot of NCI-H1975 cells treated as in Fig. 2*G* (*Upper*). Quantification of FRA1 immunoblots is shown as the mean ± SD of *N* = 3 biological replicates (*Lower*). (*D*) Immunoblot of NCI-H1975 cells treated with RBN-2397 for the indicated periods and then for 4 h with 10 μM MG132. Representative image from a single experiment with *N* = 3 biological replicates. (*E* and *F*) Immunoblots of NCI-H1975 cells following the knockdown of USP21 or PSMC3 for 48 h, respectively. Representative images from a single experiment with *N* = 3 biological replicates. Quantification of FRA1 immunoblots is indicated as the average of *N* = 3. (*G*) Immunoblot of NCI-H1975 cells following the knockdown of PSCM3 for 48 h and the treatment according to Fig. 2*G*. Representative image from a single experiment with *N* = 3 biological replicates (*Upper*). Quantification of FRA1 immunoblots is shown as the mean ± SD of *N* = 3 biological replicates (*Lower*). (*H*) Immunoblot of NCI-H1975 cells after MG132 treatment for 4 h and the knockdown of PSMC3 with two independent siRNA sequences for 48 h. Representative image from a single experiment with *N* = 3 biological replicates. Quantification of NRF2 and FRA1 immunoblots is indicated as the average of *N* = 3. (*I*) IP of PSMC3 from NCI-H1975 cells treated for 24 h with RBN-2397 and immunoblotting for FRA1. Representative image from a single experiment with *N* = 3 biological replicates. Quantification of FRA1 normalized to FRA1 input is indicated as the average of *N* = 3.

To explore how FRA1 ADP-ribosylation enhances its protein stability, we generated an ADP-ribosylation deficient FRA1 mutant (C97A). Therefore, we transduced NCI-H1975 cells with lentiviral vectors constitutively expressing FRA1-WT, C97A, or an empty vector (EV) control. After puromycin selection, single clones were picked and expanded. FRA1-C97A protein levels were substantially lower compared to the wild-type (WT) counterpart, confirming that C97 of FRA1 contributes to its stability (Fig. 3*A*). Importantly, the nuclear localization and chromatin binding capacities of FRA1-WT and C97A were highly similar, suggesting that the C97A mutation did not abrogate the molecular functions of FRA1 (*SI Appendix*, Fig. S3 *A* and *B*). Furthermore, while ADP-ribosylated FRA1-WT was pulled down from whole cell lysates using eAf1521 (28), RBN-2397 treatment, as well as the C97A mutation, prevented the pull down of FRA1, confirming that C97 serves as ADP-ribose acceptor site (Fig. 3*B*). As expected, RBN-2397 treatment did not further increase the degradation of FRA1-C97A. At the same time, FRA1-WT, like endogenous FRA1, was significantly decreased (Fig. 3*C*).

To gain a deeper mechanistic understanding of how PARP7-mediated modification of FRA1 prevents its degradation, we cotreated NCI-H1975 cells with RBN-2397 and the proteasome inhibitor MG132. Interestingly, MG132 increased baseline FRA1 levels, indicating that FRA1 is degraded by the proteasome (Fig. 3*D* and *SI Appendix*, Fig. S3*C*). In addition, we observed that proteasome inhibition for 4 h did not fully rescue FRA1 levels after prolonged (>24 h) PARP7 inhibition (Fig. 3*D* and *SI Appendix*, Fig. S3*C*), again confirming the RBN-2397-mediated degradation of FRA1. A recent report described that PSMC3 (TBP1), a 19S proteasome subunit, recruits FRA1 to the proteasome in a ubiquitin-independent manner (31). Alternatively, proteasomal degradation of FRA1 can be reversed by the ubiquitin-specific peptidase USP21 (32). To elucidate whether one of the described mechanisms regulates the turnover of FRA1, we knocked down PSMC3 and USP21, respectively, and analyzed FRA1 protein levels by immunoblotting (Fig. 3 *E* and *F*). Downregulation of USP21 only marginally affected endogenous FRA1, FRA1-WT, or C97A protein levels (Fig. 3*E* and *SI Appendix*, Fig. S3*D*). In contrast, depletion of PSMC3 substantially increased the protein levels of endogenous FRA1 and ectopically expressed FRA-WT or mutant (Fig. 3*F* and *SI Appendix*, Fig. S3*D*), suggesting that in NCI-H1975 cells, FRA1 degradation is mediated by PSMC3. Indeed, PSMC3 depletion impaired FRA1 degradation induced by RBN-2397, particularly the lower-migrating, nonphosphorylated FRA1 variants, advocating that PARP7-mediated ADP-ribosylation of FRA1 at C97 prevents its PSMC3-dependent degradation (Fig. 3*G*). Next, we tested whether the downregulation of PSMC3 increased FRA1 protein levels nonspecifically by decreasing general proteasome function. Therefore, we analyzed NRF2 protein levels after MG132 treatment and PSMC3 knockdown by immunoblotting (Fig. 3*H*). Under physiological conditions, NRF2 is constitutively expressed in cells and rapidly degraded by the proteasome (33). While proteasome inhibition by MG132 drastically increased NRF2 levels, the knockdown of PSMC3 did not stabilize NRF2 (Fig. 3*H*), suggesting that proteasome function is likely not impaired by the lack of PSMC3. Lastly, we investigated whether PARP7-mediated ADP-ribosylation would inhibit the interaction between FRA1 and the proteasome by coimmunoprecipitating PSMC3 and FRA1 from cells treated with RBN-2397. Indeed, PARP7 inhibition enhanced the complex formation between FRA1 and PSMC3 (Fig. 3*I*). Similarly, overexpression of FRA1-C97A augmented its complex formation with PSCM3 compared to FRA1-WT, confirming that ADP-ribosylation of FRA1 at C97A prevents its interaction with PSMC3 (*SI Appendix*, Fig. S3*E*). In conclusion, our data indicate that PARP7-mediated ADP-ribosylation of FRA1 inhibits the interaction of FRA1 with PSMC3 and, consequently, the degradation of FRA1 by the proteasome.

### FRA1 Regulates the Expression of Genes Involved in Apoptosis, Immune Signaling, and Cell Cycle Progression.

**Fig. 4.**
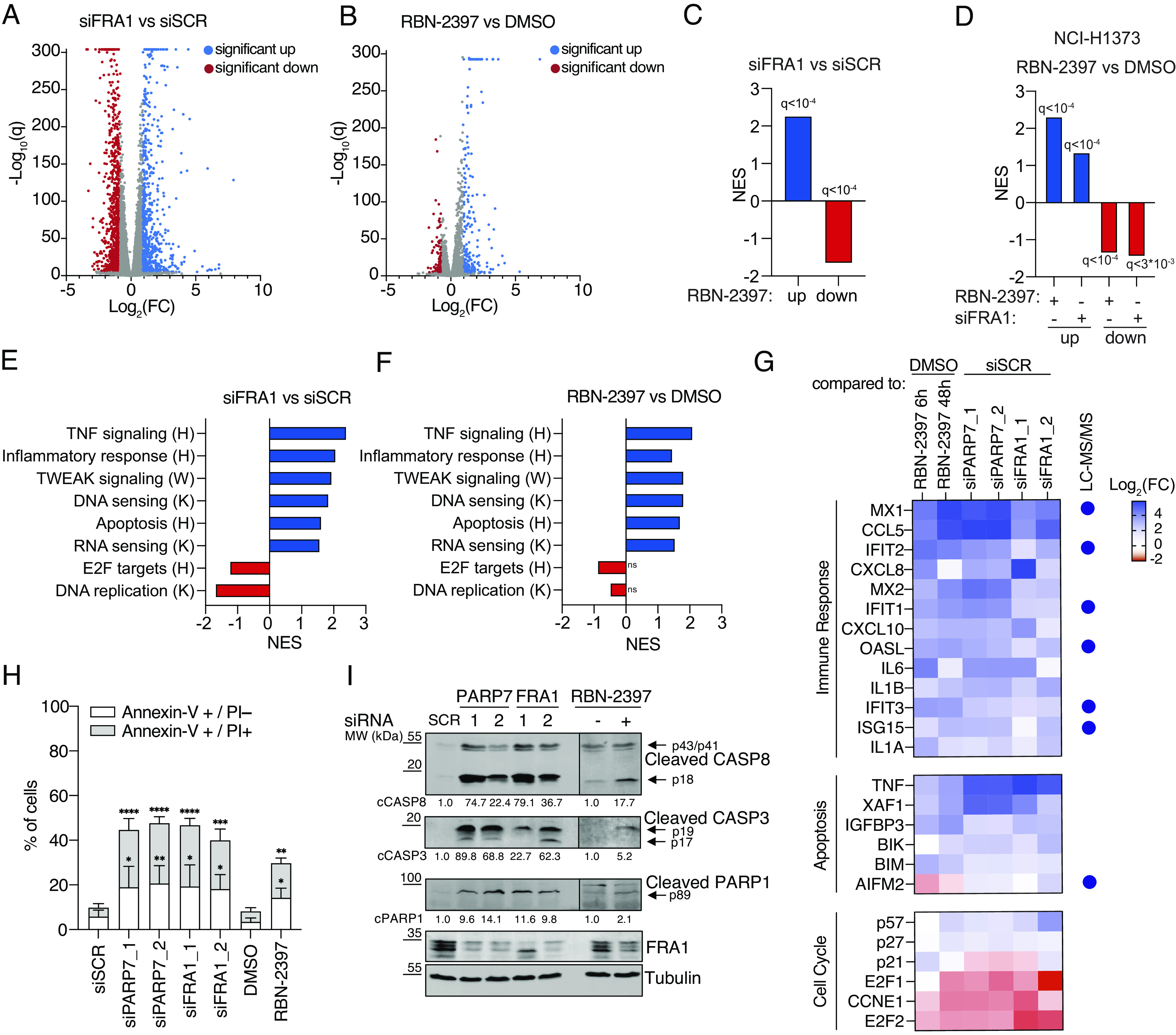
FRA1 inhibits cellular immune signaling and apoptosis and promotes cell proliferation. (*A* and *B*) Volcano plots showing changes in gene expression after 48 h FRA1 knockdown or 6 h RBN-2397 treatment in NCI-H1975 cells. Biological quadruplicates (*N* = 4) for each condition were subjected to RNA sequencing. Significant changes are indicated as FDR < 0.05 and Log_2_(FC) ≥ ±1. (*C*) Overlap between up- and down-regulated genes after siFRA1 and RBN-2397 treatment of NCI-H1975 cells. (*D*) Overlap of up- and down-regulated genes in NCI-H1373 cells after RBN-2397 treatment with differentially expressed genes in NCI-H1975 cells. (*E* and *F*) Enrichment of core gene sets after FRA1 knockdown or RBN-2397 treatment in NCI-H1975 cells. Significance as FDR-corrected *q*-values. (H: Hallmarks; K: KEGG; W: Wikipathway). (*G*) Heat map showing RT-qPCR analysis of NCI-H1975 cells after RBN-2397 treatment for 6 and 48 h and the knockdown of PARP7 and FRA1 for 48 h. Proteins significantly up-regulated in whole proteome LC–MS/MS analysis (from *SI Appendix*, Fig. S4*A*) are indicated by blue points. Data are normalized to DMSO or siSCR controls and shown as the mean of *N* = 3 biological replicates. (*H*) Bar graph showing early and late apoptotic cells following RBN-2397 treatment or PARP7 and FRA1 knockdown for 72 h in NCI-H1975 cells. Data are represented as the mean ± SD of *N* = 3 biological replicates. (*I*) Immunoblot after RBN-2397 treatment or PARP7 and FRA1 knockdown for 72 h in NCI-H1975 cells. Representative image from a single experiment with *N* = 3 biological replicates. Quantification is depicted as the mean of *N* = 3.

To elucidate how FRA1 would functionally contribute to the decrease in cell viability mediated by PARP7 inhibition, we defined the transcriptional changes in NCI-H1975 cells by RNA sequencing after siFRA1 or RBN-2397 treatment (Datasets S3 and S4). Knockdown of FRA1 resulted in the differential expression of 1732 genes (Fig. 4*A* and Dataset S3), while PARP7 inhibition led to differential expression of 310 genes (Fig. 4*B* and Dataset S4), which significantly overlapped with the up- or down-regulated genes after FRA1 depletion (Fig. 4*C*). Next, we compared our data to the published transcriptome of NCI-H1373 cells treated with RBN-2397 (5). Interestingly, we found that up- or down-regulated genes in RBN-2397 treated NCI-H1373 cells significantly overlapped with our differentially expressed genes in both siFRA1 and RBN-2397 treated NCI-H1975 cells (Fig. 4*D*). In addition, we performed whole proteome LC–MS/MS analysis of RBN-2397 treated NCI-H1975 cells and identified 162 up- or down-regulated proteins (*SI Appendix*, Fig. S4*A* and Dataset S5). Comparison of our proteomic data to the transcriptomics data revealed that the altered gene expression for siFRA1 and RBN-2397 treated cells matched the up-regulated proteins identified in the proteomics dataset (*SI Appendix*, Fig. S4*B*). To gain additional functional insight, we performed a GSEA-based pathway analysis of genes differentially expressed after FRA1 knockdown and RBN-2397 treatment and observed an enrichment of TNFα signaling, NA-sensing, apoptosis, and cell cycle genes, respectively (Fig. 4 *E* and *F*). To confirm that PARP7 activity regulates transcription via FRA1, a selected number of immune signaling, apoptotic, and cell cycle genes were analyzed by RT-qPCR after 6 h or 48 h of RBN-2397 treatment and knockdown of PARP7 or FRA1, respectively (Fig. 4*G*). As expected, we observed similar transcriptional dynamics for the tested genes. Likewise, we found identical protein expression changes within our proteomics dataset after PARP7 inhibition (Fig. 4*G*). Taken together, our data suggest that FRA1, in a PARP7-dependent manner, regulates the transcription of genes involved in immune signaling, apoptosis, and the cell cycle.

Next, we determined whether the observed differential expression of cell cycle and apoptosis genes, following FRA1 depletion and RBN-2397 treatment, could induce cell cycle dysregulation and apoptosis. Indeed, using well-accepted cell cycle and senescence markers, we observed a reduction in RB and H3 phosphorylation and an increase in β-galactosidase staining after treating cells with RBN-2397 or following PARP7 and FRA1 knockdown, respectively (*SI Appendix*, Fig. S4 *C* and *D*). Strikingly, prolonged RBN-2397 treatment or knockdown of PARP7 and FRA1 (>48 h), respectively, lead to an increased number of early (Annexin-V^+^/PI^−^) and late (Annexin-V^+^/PI^+^) apoptotic cells and promoted the robust cleavage of CASP8, CASP3, and PARP1 (Fig. 4 *H* and *I* and *SI Appendix*, Fig. S4*E*). Taken together, we demonstrated that the depletion of FRA1 induced major transcriptional changes, which resulted in reduced cell viability and was likely mediated by the activation of CASP8.

### FRA1 Suppresses Apoptosis by Inhibiting IRF1- and IRF3-Dependent RIG-I-Like Receptor Signaling.

**Fig. 5.**
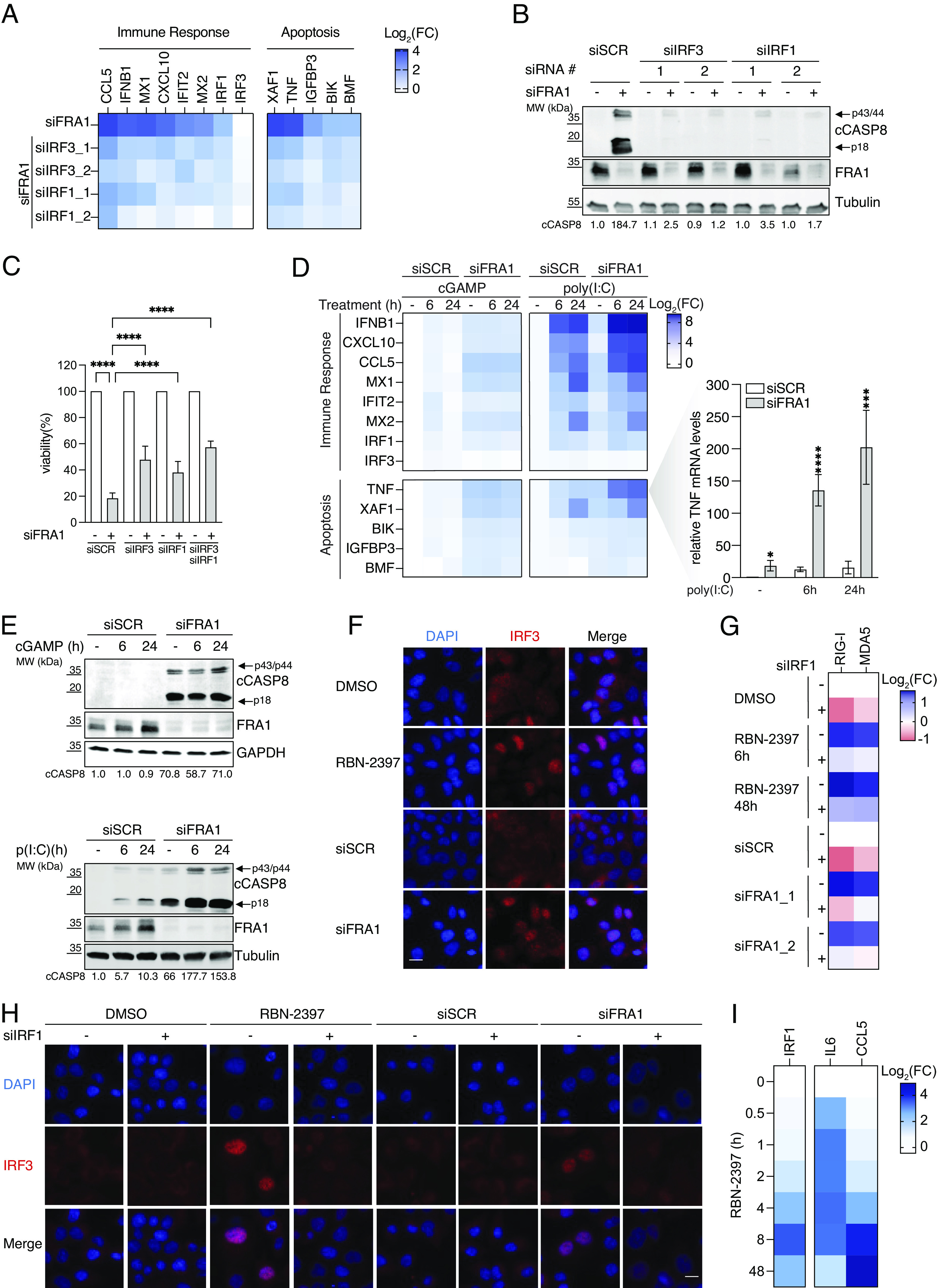
Loss of FRA1 induces IRF1- and IRF3-dependent apoptosis. (*A*) Heat map showing RT-qPCR analysis of NCI-H1975 cells, following the knockdown of FRA1 and IRF3 or IRF1 for 48 h. Data are normalized to siSCR + siSCR, siSCR + siIRF3, and siSCR + siIRF1, respectively, and shown as the mean of *N* = 3 biological replicates. (*B*) Immunoblot of NCI-H1975 cells after the cotreatment of siFRA1 with siIRF3 or siIRF1 for 48 h. Representative image from a single experiment with *N* = 3 biological replicates. Quantification is indicated as the average of *N* = 3. (*C*) Cell viability of NCI-H1975 cells following 72 h of FRA1, IRF3, and IRF1 knockdown. Data are normalized as indicated and shown as the mean ± SD of *N* = 4 biological replicates. (*D*) Heat map showing RT-qPCR analysis of NCI-H1975 cells, following the knockdown of FRA1 and the transfection of cGAMP (10 μg/mL) or poly(I:C) (10 ng/mL) and a bar graph showing detailed results for *TNF*. Data are normalized to the lipofectamine-treated siSCR control and shown as the mean ± SD of *N* = 3 biological replicates. (*E*) Immunoblot of NCI-H1975 cells treated as in *D*. Representative image from a single experiment with *N* = 3 biological replicates. Quantification is depicted as the average of *N* = 3. (*F*) IF analysis of NCI-H1975 cells after the knockdown of FRA1 or PARP7 inhibition for 48 h. Representative image from a single experiment of *N* = 3 biological replicates. The scale bar represents 20 μm. (*G*) Heat map showing RT-qPCR analysis of NCI-H1975 cells, following the knockdown of IRF1 (48 h) and the cotreatment with RBN-2397 for 6 h and 48 h or siFRA1 for 48 h. Data are normalized to siSCR + DMSO or siSCR + siSCR and depicted as the mean Log_2_(FC) of *N* = 4 biological replicates. (*H*) IF analysis as in (*F*) with additional IRF1 knockdown. Representative image from a single experiment of *N* = 3 biological replicates. The scale bar represents 20 μm. (*I*) Heat map showing RT-qPCR analysis of NCI-H1975 cells treated as in (*F*). Data are represented as the mean Log_2_(FC) of *N* = 3 biological replicates.

Given that FRA1 knockdown induces genes associated with immune signaling and apoptosis, we investigated which transcriptional regulators are critical for the immune response and CASP8-dependent apoptosis. The interferon regulatory factor 3 (IRF3) and its target gene IRF1 are critical regulators of immune signaling and apoptosis (34–38). Moreover, FRA1 was found to directly repress IRF3 activation by translocating to the cytoplasm and inhibiting TBK1 (27). Therefore, we depleted IRF3 or IRF1 and analyzed the transcriptional changes of selected FRA1 target genes by RT-qPCR. Compared to siFRA1 alone, the knockdown of FRA1 and IRF3 or IRF1 reduced both the induction of immune signaling and apoptosis-associated genes (Fig. 5*A* and *SI Appendix*, Fig. S5*A*). As expected, IRF3 was also essential for the transcriptional upregulation of IRF1 following the depletion of FRA1 (Fig. 5*A*), confirming that IRF1 is a direct target gene of IRF3. Remarkably, the lack of IRF3 or IRF1 also decreased the cleavage of CASP8 and significantly improved cell viability following the knockdown of FRA1 (Fig. 5 *B* and *C*). In addition, we also determined whether AHR, an activator of PARP7 expression (*SI Appendix*, Fig. S2*A*), contributes to apoptosis in the absence of FRA1. Simultaneous knockdown of FRA1 and AHR only slightly reduced CASP8 cleavage, despite the FRA1-dependent activation of AHR target genes *CYP1A1* and *IL1B* (*SI Appendix*, Fig. S5 *B*–*D*), suggesting that not AHR but rather IRF3 and IRF1 are necessary for the induction of apoptosis.

Next, we investigated which upstream signaling pathways promote the proapoptotic function of IRF3 and IRF1 after FRA1 knockdown. In cancer cells, aberrant cytoplasmic NAs are potent activators of cytosolic NA-sensing pathways and can promote the activation of IRF3 and IRF1 (39, 40). The binding of cellular double-stranded DNA to the cytoplasmic guanosine monophosphate-adenosine monophosphate (cGAMP) synthase (cGAS) stimulates the production of cGAMP and activates the stimulator of interferon genes (STING) (41). In addition to cGAS/STING activation, defective DNA damage responses increase aberrant cytoplasmic RNAs that trigger binding of Retinoic acid-inducible gene I (RIG-I)-like receptors (RLRs) to mitochondrial antiviral-signaling protein (MAVS) (13). Constitutive activation of STING and MAVS promotes the TBK1-dependent phosphorylation and nuclear translocation of IRF3 (42). Similarly, IRF1 can be activated by cytoplasmic DNA and RNA sensing (43, 44). Moreover, previous studies suggest that PARP7 is a critical negative regulator of cGAS/STING and RLR/MAVS signaling (5, 6). To investigate the potential proapoptotic role of NA-sensing signaling, we depleted FRA1 and transfected cells with STING and RLR agonists (cGAMP or poly(I:C)), respectively. Interestingly, we observed that RLR but not STING activation synergistically induced FRA1 target genes (i.e., *TNF*) and CASP8 cleavage (Fig. 5 *D* and *E*). Consistently, following FRA1 knockdown, cells treated with the cGAS inhibitor G140 only slightly reduced the transcriptional upregulation of FRA1 target genes and did not prevent apoptosis via the cleavage of CASP8 (*SI Appendix*, Fig. S5 *E* and *F*), indicating that RLR-signaling contributes most significantly to apoptosis in NCI-H1975 cells. Given the substantial upregulation of *TNF* after FRA1 depletion, we investigated whether the secretion of TNFα would initiate apoptosis in a paracrine manner by activating complex IIa (45). Although we observed that treatment with exogenous TNFα synergized with the knockdown of FRA1 in inducing CASP8-dependent apoptosis (*SI Appendix*, Fig. S5 *G* and *H*), blocking endogenous TNFα with a neutralizing antibody did neither rescue the cleavage of CASP8 nor cell viability after the knockdown of FRA1 (*SI Appendix*, Fig. S5 *G* and *H*). Together, these data suggest that increased TNFα expression following PARPP7 inhibition is not the initiator of apoptosis but promotes its amplification and that the activation of CASP8 upon FRA1 loss is TNFα independent.

Next, we investigated how FRA1 exerts its repressive function toward the RLR-signaling-dependent activation of IRF3/IRF1. In contrast to a previous report (27), neither poly(I:C) treatment nor PARP7 inhibition induced a cytoplasmic translocation of FRA1 (*SI Appendix*, Fig. S5*I*), suggesting an alternative mechanism for the FRA1-dependent inhibition of IRF3. Remarkably, we found that both FRA1 knockdown and PARP7 inhibition, comparable to poly(I:C) transfection, promoted the nuclear translocation of IRF3 (Fig. 5*F* and *SI Appendix*, Fig. S5*J*), indicating that nuclear FRA1 indirectly exerts its repressive function toward cytoplasmatic IRF3. A previous study suggested that IRF1-dependent upregulation of *DDX58* (RIG-I) and *IFIH1* (MDA5) leads to the activation of IRF3 and, thus, inflammatory and proapoptotic gene expression (38). Consistent with these observations, we found that upregulation of RIG-I and MDA5 after PARP7 inhibition and FRA1 depletion was dependent on IRF1 (Fig. 5*G*), suggesting that FRA1 represses IRF3 activation by inhibiting the IRF1-dependent RIG-I and MDA5 upregulation. Indeed, the depletion of IRF1 not only dampened the increase in RIG-I and MDA5 expression but also reduced the nuclear translocation of IRF3 following RBN-2397 and siFRA1 treatment (Fig. 5*H*) without abrogating the NA-dependent activation of RLR signaling (*SI Appendix*, Fig. S5*K*). Noteworthy, in comparison to immediate response genes like *IL6,* we found that *IRF1* was transcriptionally increased only after extended PARP7 inhibition (Fig. 5*I*). Therefore, suggesting that, under basal conditions, FRA1 blocks the IRF1-dependent transcription of RIG-I and MDA5 and that IRF1 expression likely increases only after the activation of IRF3 (Fig. 5*A*). Together, these findings provide strong evidence that FRA1 functions as a negative regulator of IRF1, which suppresses an RLR-signaling and IRF3-dependent feedforward loop that ultimately inhibits immune signaling and apoptosis.

### PARP7 Expression Is a Marker for RBN-2397 Sensitivity of FRA1-Driven Cancer Cells.

**Fig. 6.**
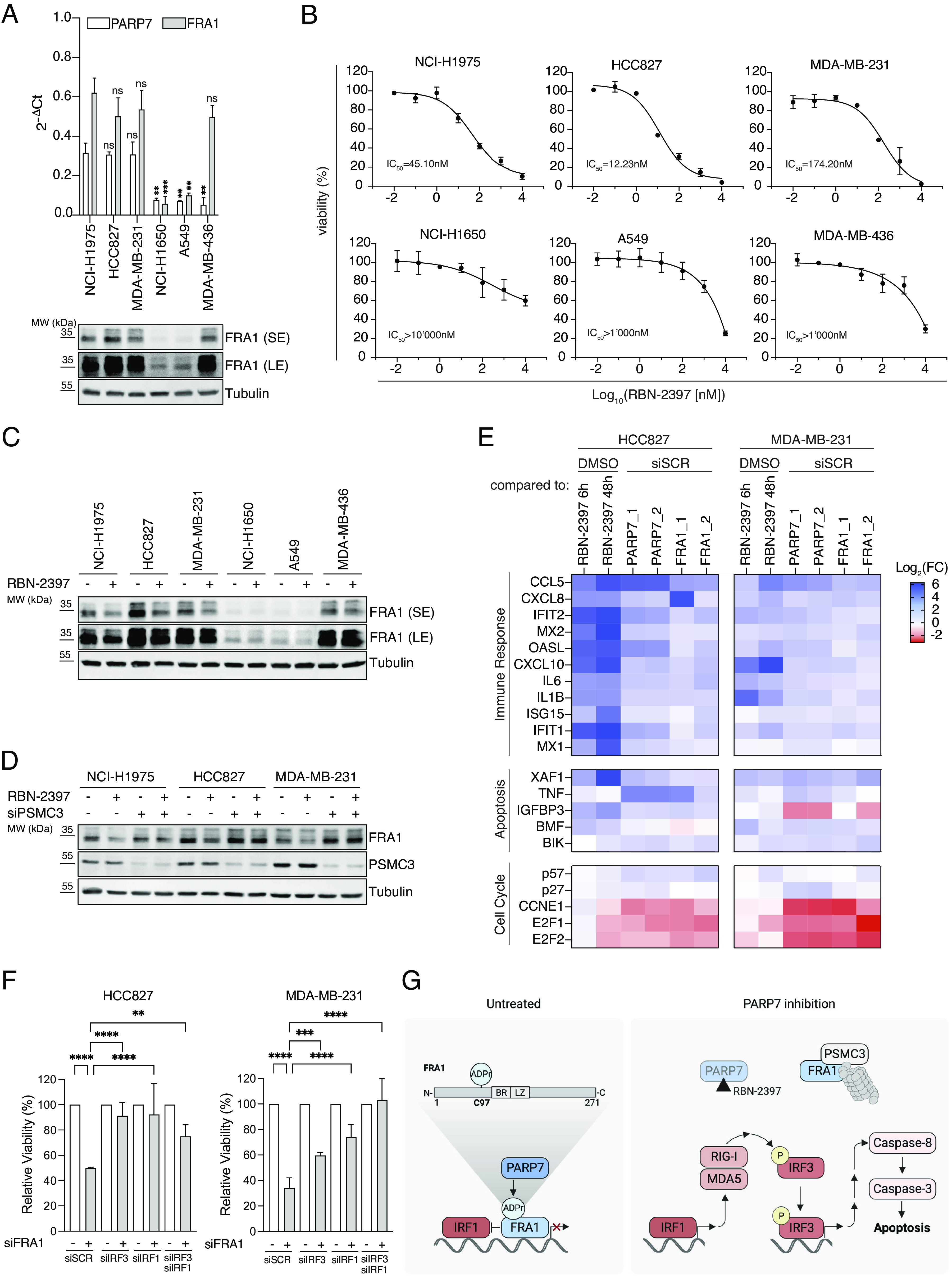
Cellular RBN-2397 sensitivity is dependent on high PARP7 expression in FRA1-positive cancer cell lines. (*A*) RT-qPCR analysis (*Upper*) and immunoblot (*Lower*) are shown for the indicated cell lines and depicted as the mean ± SD of *N* = 2 biological replicates. Representative immunoblot from a single experiment with *N* = 3 biological replicates (SE: short exposure; LE: long exposure). (*B*) Cell viability of indicated cell lines following six days of treatment with increasing concentrations of RBN-2397. Data are depicted as the mean ± SD of *N* = 2 biological replicates. Curves were fitted using a four-parametric nonlinear model. (*C*) Immunoblot following RBN-2397 treatment for 72 h in the indicated cell lines. Representative image from a single experiment with *N* = 3 biological replicates (SE: short exposure; LE: long exposure). (*D*) Immunoblot following RBN-2397 treatment for 72 h and PSCM3 knockdown for 48 h in the indicated cell lines. Representative image from a single experiment with *N* = 3 biological replicates. (*E*) Heat map showing RT-qPCR analysis of HCC827, and MDA-MB-231 cells treated as in Fig. 4*G*. Data are normalized to DMSO or siSCR controls and shown as the mean of *N* = 2 biological replicates. (*F*) Cell viability following 72 h days of knockdown. Data are depicted as the mean ± SD of *N* = 4 biological replicates. (*G*) Schematic of the proposed mechanism of action of RBN-2397 in FRA1-driven lung and breast cancer cells. Under untreated conditions, FRA1 is ADP-ribosylated by PARP7 on C97 (BR, basic region; LZ, leucine zipper). Loss of FRA1 ADP-ribosylation by PARP7 inhibition results in the PSMC3-dependent proteasomal processing of FRA1. The degradation of FRA1 increases the IRF1-dependent expression of RIG-I and MDA5, which in turn promotes the activation and nuclear translocation of IRF3. The activation of IRF3 allows for the upregulation of cytokine expression and promotes CASP8-dependent apoptosis.

To investigate whether the identified PARP7/FRA1/IRF1/IRF3 axis is observed in other cancer cell lines, we compared FRA1 and PARP7 expression levels across 1,078 cell lines from various origins using the DepMap project dataset. Higher FRA1 mRNA levels corresponded with higher FRA1 cell line dependency (*SI Appendix*, Fig. S6*A*). Of note, a recent report found that PARP7 mRNA levels positively correlated with PARP7 cell dependency (5). Based on these observations, we explored whether high FRA1 expression levels would indicate PARP7 dependency by comparing FRA1 expression levels with the dependency scores of all assessed genes in the DepMap database. Among the top two genes exhibiting a substantial correlation with FRA1 expression, we identified FRA1 and PARP7, suggesting that high FRA1 expression levels correlated with a higher PARP7 dependency (*SI Appendix*, Fig. S6 *B* and *C*). Thus, we determined whether other lung as well as breast cancer cell lines that express high levels of FRA1 and/or PARP7 also exhibited increased RBN-2397 sensitivity compared to cell lines with lower FRA1 and/or PARP7 expression levels (Fig. 6*A* and *SI Appendix*, Fig. S6*D*). Indeed, RBN-2397 decreased the viability of cell lines expressing higher levels of FRA1 and PARP7 in a dose-dependent manner, with IC_50_ values comparable to those observed in NCI-H1975 cells (Fig. 6*B*). In contrast, all other tested cell lines were insensitive to RBN-2397, including MDA-MB-436 cells which have very little PARP7 but express FRA1 at levels similar to all of the RBN-2397-sensitive cells (Fig. 6 *A* and *B* and *SI Appendix*, Fig. S6*D*). Together, these findings indicate that PARP7 levels can be used to predict the RBN-2397 sensitivity of FRA1-positive lung and breast cancer cells.

To confirm that PARP7 inhibition leads to FRA1 degradation in all RBN-2397 sensitive cell lines, we treated cells with the PARP7 inhibitor for 72 h and analyzed FRA1 protein turnover by immunoblotting. All RBN-2397 sensitive cell lines showed a significant decline in FRA1 protein levels (Fig. 6*C* and *SI Appendix*, Fig. S6*E*). In contrast, FRA1 protein levels did not decrease in the insensitive cell lines, suggesting that FRA1 is not controlled by PARP7s’ enzymatic activity in these cells (Fig. 6*C* and *SI Appendix*, Fig. S6*E*). Next, we verified whether PARP7-mediated ADP-ribosylation of FRA1 would also inhibit its proteasomal degradation in the sensitive cell lines. Indeed, the knockdown of PSMC3 rescued the RBN-2397-dependent degradation of FRA1 in all sensitive cell lines, confirming that in RBN-2397-sensitive cell lines PARP7 stabilizes FRA1 and inhibits its degradation by PSMC3 (Fig. 6*D* and *SI Appendix*, Fig. S6*F*). Similarly, RBN-2397 treatment and PARP7 or FRA1 knockdown increased the expression of inflammatory and apoptotic genes (Fig. 6*E* and *SI Appendix*, Fig. S6 *G* and *H*). At the same time, we observed a reduction in the proliferative signature of HCC827 and MDA-MB-231 cells (Fig. 6*E*); further validating our results in NCI-H1975 cells following PARP7 and FRA1 depletion (Fig. 4*G*). Lastly, we observed that IRF3 and IRF1 knockdown in HCC827 and MDA-MB-231 cells significantly improved cell viability following FRA1 downregulation (Fig. 6*F*). Collectively, our findings suggest that PARP7 inhibition induces IRF1- and IRF3-dependent apoptosis by promoting the degradation of FRA1 in FRA1-driven lung and breast cancer cell lines (Fig. 6*G*).

## Discussion

In recent years, combination therapies, which harness the synergistic effects of immune checkpoint inhibitors and intratumoral innate immunity, have emerged as promising strategies to control tumor development and progression (46). Considerable attention has been given to PARP7, mainly because its inhibition restores type I IFN signaling in cancer cells and results in durable, complete tumor regression in human cancer xenografts and clinical trials (15). Moreover, the PARP7 inhibitor RBN-2397 was also shown to inhibit cancer cell growth in a cell-autonomous manner by regulating cell death and cell proliferation (5, 47). However, the mode of action of PARP7 inhibitors, as well as the identification and validation of PARP7 targets and, thus, potential biomarkers that suppress cancer cell immune signaling and prompt cancer cell viability, have been missing. To address this issue, we explored PARP7 activity in PARP7 inhibitor-sensitive lung and breast cancer cell lines using an integrated approach that combined MS-based ADP-ribosylome analyses with transcriptomics and proteomics.

Here, we identified endogenous PARP7 targets using an LC– MS/MS-based enrichment strategy of ADP-ribosylated proteins by comparing the modified peptides identified in the presence or absence of PARP7 inhibition (28). We found that endogenous PARP7 predominantly localizes to the nucleus and modifies its targets solely on cysteine residues. Among the PARP7 target proteins, we identified the AP-1 transcription factor FRA1 as essential for cell survival and a crucial regulator of PARP7 inhibitor-mediated cell death. Our data suggest that PARP7-mediated ADP-ribosylation of FRA1 at C97 prevents the binding of FRA1 to PSMC3, a 19S proteasome subunit, and thus its proteasomal degradation. Moreover, comparable to the increase in FRA1 stability mediated by PARP7 ADP-ribosylation, it was previously reported that FRA1 is stabilized by phosphorylation at its C-terminus (20, 25, 48). However, we observed that the stabilization of FRA1 by PARP7 is regulated independently of FRA1’s phosphorylation. Hence, we hypothesize that PARP7-mediated ADP-ribosylation specifically regulates FRA1 binding to PSMC3 and, thus, FRA1 degradation.

In contrast to a previous report, AHR was not ADP-ribosylated in NCI-H1975 cells (9). Nevertheless, we confirmed that AHR expression sensitizes cells to PARP7 inhibition (47). Therefore, further investigations are required to understand how AHR sensitizes cells toward PARP7 inhibition, independent of AHR ADP-ribosylation. Furthermore, we found that AHR partly controls PARP7 expression and that FRA1 inhibits the upregulation of a subset of AHR target genes (e.g., *CYP1A1*). These findings point toward a complex interplay between AHR, PARP7, and FRA1, in which PARP7-mediated ADP-ribosylation of FRA1 might impair AHR-dependent transcription.

Consistent with previous reports describing PARP7 as a negative regulator of innate immune signaling, we found that FRA1 represses the expression of genes associated with innate immunity (5, 6). However, we did not detect the previously described PARP7-mediated ADP-ribosylation of TBK1 (5, 49). In addition, our results suggest that PARP7 exclusively localizes to the nucleus, whereas TBK1 localizes to the cytoplasm. Therefore, our findings support the conclusion of a previous study demonstrating that PARP7 inhibition regulates type I IFN signalling downstream of TBK1 (6). Remarkably, in the absence of FRA1, we found that the downstream target of TBK1, IRF3, and its target gene IRF1 were crucial transcription factors for the induction of immune signaling and apoptosis. While IRF3 activation was solely dependent on RLR-signaling in NCI-H1975 cells, other studies found the cGAS/STING pathway to induce IRF3-dependent immune signaling across various RBN-2397 sensitive cell lines (5, 6). These findings emphasize the critical and central role of IRF3 activation in inducing apoptosis in cancer cells following PARP7 inhibition, independent of the cell-type specific upstream signaling (i.e., RLR or cGAS/STING signaling). Moreover, we found that the repressive function of FRA1 toward IRF3 is indirect since IRF3 was only activated and, in turn, translocated to the nucleus in an IRF1-dependent manner after the loss of FRA1. Consistent with a previous report (38), we found that IRF3 was initially activated through the IRF1-dependent upregulation of RIG-I and MDA5, which suggests a feedforward loop in which IRF1 activates IRF3 and IRF3 in turn transcriptionally up-regulates IRF1. Based on our data, we cannot conclusively exclude that PARP7 inhibition through an uncharacterized mechanism would also increase aberrant cytoplasmic NA and thereby activate IRF3. In conclusion, our findings highlight how the PARP7-FRA1 axis regulates IRF1- and, consequently, IRF3-mediated cell intrinsic apoptosis signaling in lung and breast cancer cells.

FRA1 is regarded as a potent oncogene, and its overexpression is associated with more malignant tumors and poor patient outcomes (20). Intriguingly, we could demonstrate that high PARP7 expression levels are critical for RBN-2397 sensitivity of FRA1-positive lung and breast cancer cell lines and FRA1 protein stability. Based on this finding, we hypothesize that assessing PARP7 expression levels might be of clinical importance for most FRA1-positive cells, especially since the PARP7 inhibitor RBN-2397 has entered clinical trials (15).

## Materials and Methods

Detailed methods are provided in supporting information. Human cell lines were obtained from ATCC or were a gift from Ursula Klingmüller (DKFZ, Heidelberg, Germany) and verified regularly for Mycoplasma infection. NCI-H1975, NCI-H1650, MDA-MB231, MDA-B436, A549, and HEK293T cells were cultured in high glucose-containing Dulbecco’s modified Eagle’s medium (DMEM); HCC827 cells were cultured in high glucose RPMI-1640 medium. All media were supplemented with 10% fetal bovine serum and 1% penicillin/streptomycin. All cells were grown at 37 °C in a humidified atmosphere with 5% CO_2_.

## Supplementary Material

Appendix 01 (PDF)

Dataset S01 (XLSX)

Dataset S02 (XLSX)

Dataset S03 (XLSX)

Dataset S04 (XLSX)

Dataset S05 (XLSX)

Dataset S06 (XLSX)

## Data Availability

Proteomic and sequencing data have been deposited in ProteomeXchange (PXD041870) (50) and GEO (GSE229674) (51). Previously published data were used for this work [We used the publicly accessible transcriptomics data set GSE177494 (RBN-2397 treatment of NCI-H1373 cells for 24 h). The RNA sequencing data of 1,078 cell lines, cell line annotations, and gene dependency scores were downloaded from the portal of the Dependency Map (DepMap) project (https://depmap.org/portal, release: Public 22Q4)] (52).
