# Supplementary material for: PARP7-mediated ADP-ribosylation of FRA1 promotes cancer cell growth by repressing IRF1- and IRF3-dependent apoptosis
Source: Proc Natl Acad Sci U S A. 2023 Nov 27;120(49):e2309047120. doi: 10.1073/pnas.2309047120 (PMC10710093; doi:10.1073/pnas.2309047120)
Supplement: Supplementary file 1 — Appendix 01 (PDF) [file pnas.2309047120.sapp.pdf]

Supporting Information for

PARP7-mediated ADP-ribosylation of FRA1 promotes cancer cell growth by repressing IRF1- and IRF3-dependent apoptosis

Patrick Manetsch, Flurina Böhi, Kathrin Nowak, Deena M. Leslie Pedrioli and Michael O. Hottiger

Corresponding Author: Michael O. Hottiger
Email: michael.hottiger@dmmd.uzh.ch

This PDF file includes:

Supporting Material and Methods
Figures S1 to S6
Legends for Datasets S1 to S6
SI References

Other supporting materials for this manuscript include the following:

Datasets S1 to S6

Supporting Information - Text

Material and Methods

Cell culture

For PARP7 inhibition, all cell lines were treated with 100nM RBN-2397 (MedChemExpress LLC, Monmouth Junctions, NJ), if not stated otherwise. NCI-H1975 cells stably expressing empty vector (EV), WT, or C97A FRA1 were cultured in a complete DMEM medium supplemented with 0.5μg/ml Puromycin. A549 cells stably expressing PARP7 were cultured in a complete DMEM medium supplemented with 30μg/ml Blasticidin, and PARP7 expression was induced using 250 ng/ml Doxycycline for 48h.

FRA1 overexpression in NCI-H1975 cells

The FRA1 coding sequence (Addgene plasmid #34847) was subcloned into a lentiviral vector (Addgene plasmid #19311). The FRA1 C97A mutation was generated by site-directed mutagenesis. Lentiviruses were produced by co-transfecting HEK293T cells with viral EV or FRA1 constructs, psPAX2, and pVSVG plasmid (Addgene plasmids #12260 and #12259) in a ratio of 1:1 (construct / packaging and envelope) and 8:1 (packaging / envelope) using calcium phosphate. 6h after transfection, the medium was removed and replaced with fresh medium. 2 days after transfection, the medium was collected and filtered through a 0.45μm membrane filter to eliminate residual cells. Approximately 40% of confluent NCI-H1975 cells were transduced with the virus-containing medium supplemented with 5 mg/mL polybrene. 24h after transduction, cells were subjected to puromycin selection (1μg/ml). Following the antibiotic selection (~72h), cells were expanded as single clones in puromycin selection medium (0.5 μg/ml).

PARP7 overexpression in A549 cells

The transposase-mediated stable integration of PARP7 was performed by co-transfecting A549 cells with the PARP7 plasmid and the Sleeping Beauty Transposase (Addgene plasmid #34879) using Lipofectamine 3000 (Thermo Fisher). The PARP7 coding sequence was subcloned into the Sleeping Beauty vector (Addgene plasmid #60506). 72h after transfection, cells were subjected to Blasticidin selection.

Endogenous tagging of PARP7 in NCI-H1975 cells

As previously described, a 3xFLAG-tag was knocked-in into the *PARP7* gene at the C-terminus (*1*). The sgRNA targeting the C-terminus of *PARP7* (5'-ttaaaaatgacaaaaatctg -3') was cloned into the sgRNA scaffold (Addgene plasmid #48138). NCI-H1975 cells were transfected with the sgRNA plasmid, the Cas9 encoding plasmid (Addgene plasmid #6694), and the 3xFLAG-Puro plasmid (Addgene plasmid #80973) using Lipofectamine 3000 (Thermo Fisher). After 72h, cells were subjected to puromycin selection. IF and knockdowns of PARP7 verified the integration of the FLAG tag.

cGAMP and poly(I:C) transfection

For the transfection of cGAMP (Invivogen) and low molecular weight polyinosinic-polycytidylic acid (poly(I:C) (Invivogen), NCI-H1975 cells were seeded at 90% confluency in 6-well plates. The following day, cells were transfected with 10μg/ml cGAMP or 10ng/ml poly(I:C) using Lipofectamine 3000 (Thermo Fisher) for the indicated time. In addition, cells were treated with Lipofectamine 3000 alone as a control.

siRNA transfection

siRNA-mediated knockdowns were performed via reverse transfection using Lipofectamine RNAiMAX (Thermo Fisher). On the day of the transfection, 10nM siRNA were mixed with 5μl lipofectamine in 500μl serum-free OptiMEM and incubated for 20min at room temperature (RT). Then, siRNA was added dropwise to cell suspensions in 6-well plates. To avoid cytotoxicity, the medium was replaced 12h following the transfection. A scrambled siRNA (siSCR) was used for each experiment as a control. For co-knockdowns, the total siRNA concentration was 10nM. The time of treatment is indicated in the figure legends.
Target sequences for siRNA-mediated knockdowns are listed in Dataset S6.

siRNA screen
siRNA-mediated knockdowns were performed via reverse transfection using Lipofectamine RNAiMAX (Thermo Fisher). On the day of the transfection, 5nM siRNA was distributed onto a 96-well plate in a volume of 10µl. Afterwards, 40µl serum-free OptiMEM premixed with 1.6µl lipofectamine per reaction was added and mixed, and the reaction was subsequently incubated for 20 min at RT. After the incubation, 150µl of cell suspension was added to reach 1000 cells per well. Immediately following the transfection, DMSO or RBN-2397 (100nM; purchased from MedChemExpress LLC, Monmouth Junctions, NJ) pre-diluted in medium was added to the cells. A siSCR was used as a control for each experiment, and siSCR was present on three positions on each plate to control for plate-to-plate variability. 5 days after the transfection, the cell viability assay was performed as described below. Data are normalized using the global mean of siSCR. Viability values from three biological replicates were averaged, and each siRNA's sensitivity index (SI) was determined as previously described (*2*). SI scores were standardized using a robust z-transformation (MAD z-score) to ensure a normal distribution. Genes were selected as hits if two out of three siRNA were below the threshold of -1.5.
Target sequences for siRNA-mediated knockdowns are listed in Dataset S6.

Cell viability assay
To determine cell viability, 1000 cells were seeded as technical sextuplicate (siRNA transfection) or 12-plicates (RBN-2397 sensitivity) in a 96-well (Costar). Following drug treatment or siRNA transfection, cell viability was measured using a CellTiter-Blue assay (Thermo Fischer). Cells were washed with PBS and incubated in 100µl complete medium containing 0.015 mg/ml resazurin sodium salt. Fluorescence was recorded at 560 nm excitation with a 590 nm emission filter. Blank values were subtracted, and data were averaged across technical replicates and normalized to the DMSO or siSCR control. To determine RBN-2397 sensitivity, four-parametric non-linear dose-response curves were fitted using GraphPad Prism to infer half-maximal growth-inhibitory concentrations (IC_{50}).
Antibodies and compounds used for viability analysis: TNF-α neutralizing antibody (#7321, Cell Signaling, 1ug/ml), TNF-α (300-01A PeproTech, 100ng/ml).

Immunoblotting
For IB analysis, cells were lysed in SDS lysis buffer (60mM Tris/HCL pH=8, 2% SDS, and 10% Glycerol), and lysates were denatured by heating at 70°C for 3 min, followed by sonication. Proteins were separated via SDS page on a 12%-SDS-polyacrylamide gel at 120V. The wet transfer onto a PVDF membrane was performed at 100 V for 2h, and membranes were then blocked with 5% milk in TBS-T for 1h at RT. Primary antibodies were diluted in 5% milk (TBS-T) and incubated at 4°C overnight. After four washes, the secondary antibody, diluted in 5% milk, was incubated with the membrane for 1h at RT. After another four washes, specific proteins/bands were visualized with the Odyssey infrared imaging system (LI-COR). IB was quantified using FIJI, and protein levels were normalized to the GAPDH or Tubulin loading control, respectively.
Primary and secondary antibodies used for IB analysis: FRA1 (#5281, Cell Signaling, 1:1000), pFRA1 (#5841, Cell Signaling, 1:1000), PSMC3 (#13923, Cell Signaling, 1:1000), c-JUN (#9165, Cell Signaling, 1:1000), FLAG (F1804, Sigma Aldrich, 1:2000), HA (ab9110, Abcam, 1:5000), GAPDH (#5147, Cell Signaling, 1:1000), α-Tubulin (T6199, Sigma Aldrich, 1:5000), H3 (#4499, Cell Signaling, 1:5000), pH3 (#9701, Cell Signaling, 1:1000), RB (#2655, Epitomics, 1:500), pRB (#8516, Cell Signaling, 1:1000), cleaved CASP3 (#9661, Cell Signaling, 1:1000), cleaved CASP8 (#9496, Cell Signaling, 1:1000), cleaved PARP1 (#9546, Cell Signaling, 1:2000), IRF3 (#4302, Cell Signaling, 1:500), STAT1 (#9172, Cell Signaling, 1:1000), pSTAT1 (33-3400, Thermo Fischer, 1:500), NRF2 (PA5-27882, Thermo Fisher, 1/1000).
Secondary antibodies used for IB analysis: goat anti-mouse 800CW (#926- 32210, LI-COR Biosciences, 1:15000), donkey anti-rabbit 680CW (#926-68023, LICOR Biosciences, 1:15000).

Immunofluorescence (IF) analysis

For IF experiments, cells were grown on glass coverslips. After treatment, cells were fixed with 4% formaldehyde (in PBS) for 20 min at RT and permeabilized for 8 min at RT in PBS supplemented with 0.2% Triton X-100 (Sigma Aldrich). After blocking the cells in the blocking solution (PBS supplemented with 2% bovine serum albumin) for 2 h, the cells were incubated with the primary antibody (diluted in blocking solution) overnight at 4 °C. Next, coverslips were washed three times with PBS and subsequently incubated with the secondary antibody (diluted in blocking solution) for 1h at RT. Afterwards, the cells were incubated with 0.1 µg/ml DAPI in PBS for 20 min at RT. Coverslips were washed three times with PBS for 5 min and mounted on glass slides using Mowiol® 4-88. Images were taken with a fluorescence microscope (Leica Thunder). For all images, brightness and contrast were adjusted using FIJI. The same acquisition and image processing settings were used for all images within one experiment, except stated otherwise.

Primary and secondary antibodies used for IF analysis: FRA1 (#5281, Cell Signaling, 1:1000), FLAG (F1804, Sigma Aldrich, 1:500), HA (ab9110, Abcam, 1:1000), eAf1521 (homemade, 1:200), IRF3 (ab68481, Abcam, 1/100).

Secondary antibodies used for IF analysis: goat anti-rabbit IgG(H+L) Alexa Fluor 647 (A21244, Invitrogen, 1:500), goat anti-mouse rabbit IgG(H+L) Alexa Fluor 488 (A11029, Invitrogen, 1:500).

β-galactosidase staining

For β-galactosidase staining, cells were seeded in 6 well plates and left to adhere overnight. Cells were treated with 100nM RBN-2397 or siRNAs targeting PARP7 and FRA1 for 48h, and β-galactosidase activity was measured using the Senescence β-Galactosidase Staining Kit (Cell Signaling Technology, #9860) according to the manufacturer's instructions. Images were taken using a 10X Brightfield Microscope (Olympus) and the EOS 800D camera (Canon).

RT-qPCR

Total RNA was isolated using the NuceloSpin kit (Macherey-Nagel) according to the manufacturer's instructions, including DNase digestion. 2µg of total RNA was reverse transcribed using a MultiScribe Reverse transcriptase kit (Applied Biosystems) with random hexamer primers. Quantitative real-time PCR (qPCR) was performed using QuantStudio5 System (Applied Biosystems) with the SYBR Fast qPCR Master Mix (Sigma Aldrich). qPCR primers used to analyze mRNA and pre-mRNA levels are listed in Dataset S6. Data are normalized to the expression of GAPDH, and the difference between treated vs. untreated cells was calculated as ΔΔCt. For visualization, fold changes were calculated as $2^{-\Delta\Delta Ct}$ or $-Log_2(2^{-\Delta\Delta Ct})$.

Chromatin fractionation

To assess the chromatin binding of WT and C97A FRA1, cells were lysed in chromatin extraction buffer (10mM HEPES, 100nM NaCl, 2mM $MgCl_2$, 0.5% Triton-X 100, protease inhibitors). 10% of the lysate was kept aside as a whole cell lysate (WCL) control. The remaining lysates were incubated at 4 °C under constant rotation for 30 min and then centrifuged at 14,000× g for 10 min at 4 °C. The resulting pellet was resuspended in chromatin extraction buffer and sonicated, and the supernatant and the WCL were analyzed by immunoblotting for H3 (chromatin control), tubulin (supernatant control), and FRA1.

Co-immunoprecipitation (IP)

NCI-H1975 cells were treated with RBN-2397 for 24h. The following day cells were lysed in a buffer maintaining protein-protein interactions (50mM Tris/HCl pH=8, 100mM NaCl, 2mM $MgCl_2$, 10% Glycerol, 1% NP-40, protease inhibitors) and incubated at 4 °C under constant rotation for 30 min. The lysates were then clarified by centrifugation at 14,000× g for 10 min at 4 °C. For the IP of WT and C97A FRA1, the lysates were incubated with anti-FLAG Sepharose beads (previously equilibrated in wash buffer, Sigma Aldrich) for 2h at 4 °C. For the IP of PSMC3 the lysates were incubated with an antibody against PSCM3 (1:50, Cell Signaling #13923) or an IgG-rabbit antibody (Millipore) as a control for 2h at 4 °C. Next, protein G Sepharose beads were equilibrated in wash buffer (50mM Tris/HCl pH=8, 100mM NaCl, 2mM $MgCl_2$, 10%Glycerol, 0.1% NP-40, protease inhibitors) and added to the IP reaction for 1h. After the incubation, all IP reactions were washed three times with a wash buffer. At the end of the washing steps, the resins were resuspended in 30µl SDS sample buffer, boiled, and analyzed by immunoblotting for FRA1 or PSCM3.

eAf1521-pull down for immunoblotting

eAf1521-pull down was performed as previously described (*3*). Following RBN-2397 treatment for 24h, NCI-H1975 cells overexpressing WT or C97A FRA1 were lysed in modified RIPA buffer (100 mM Tris HCl, pH 7.5, 150 mM NaCl, 1% NP40, 0.3% deoxycholate, 0.1% SDS, protease inhibitors, 5μM PJ34) and incubated at 4°C under constant rotation for 30min. Three times more FRA1-C97A expressing cells were used and lysed in the same volume as FRA1-WT expressing cells The lysates were then clarified by centrifugation at 14,000× g for 10min at 4°C; the resulting supernatants were incubated with 10μg of GST-tagged eAf1521 or the binding deficient mutant eAf1521 G42E. After overnight incubation at 4°C, GSH resin (previously equilibrated with RIPA buffer) was added for an additional hour to recover eAf1521-bound proteins. Then, samples were centrifuged at 500×g for 2min and washed three times in RIPA buffer. At the end of the washing steps, the resins were resuspended in 30μl SDS sample buffer, boiled, and analyzed by immunoblotting for FLAG.

Apoptosis analysis by flow cytometry

For apoptosis analysis, cells were treated with RBN-2397 or siRNAs targeting PARP7 and FRA1 for 72h. Supernatants and cells were harvested and stained with Annexin-V and PI using the Abcam apoptosis detection kit (ab14085). Apoptotic cells were analyzed on a BD FACSCanto (BD biosciences), and Annexin-V+ / PI- and Annexin-V+ / PI+ cells, excluding cell doublets, were determined using FlowJo.

RNA sequencing and GSEA analysis

Total RNA was extracted using the RNAeasy Plus Mini Kit (Qiagen). The quality of the isolated RNA was determined with a Fragment Analyzer (Agilent). The TruSeq Stranded mRNA kit (Illumina) was used in the subsequent steps. Briefly, total RNA samples (100-1000ng) were poly-A enriched and then reverse-transcribed into double-stranded cDNA. The cDNA samples were fragmented, end-repaired, and adenylated before the ligation of TruSeq adapters containing unique dual indices (UDI) for multiplexing. Fragments containing TruSeq adapters on both ends were selectively enriched with PCR. The quality and quantity of the enriched libraries were validated using the Fragment Analyzer (Agilent). The product is a smear with an average fragment size of approximately 260bp. The libraries were normalized to 10nM in Tris-HCl (10 mM, pH=8.5) with 0.1% Tween20. According to standard protocol, the Novaseq 6000 (Illumina) was used for cluster generation and sequencing. The sequencing configuration was single-end 100bp. For the analysis, demultiplexing was performed using the Illumina bcl2fastq Conversion Software. Individual library sizes ranged from 22.5 million to 39.2 million reads. RNA sequencing analysis was performed using the SUSHI framework (*4*), encompassing the following steps: Read quality was inspected using FastQC, and sequencing adaptors were removed using fastp; Alignment of the RNA-Seq reads using the STAR aligner (*5*) and with the GENCODE human genome build GRCh38.p13 (Release 37) as the reference (*6*); the counting of gene-level expression values using the 'featureCounts' function of the R package Rsubread (*7*); differential expression using the generalized linear model as implemented by the DESeq2 Bioconductor R package (*8*); GO term pathway analysis using both the hypergeometric over-representation test via the 'enricher' function and gene-set enrichment analysis via the 'GSEA' function, of the clusterProfiler Bioconductor R package (*9*). For the GSEA-based pathway analysis, we included gene sets from the MSigDB Hallmark, KEGG, and Wiki pathways data bases. All R functions were executed on R version 4.1.2 and Bioconductor version 3.14.

ADPr-peptide enrichment

Cells were lysed in lysis buffer (6M GndHCl, 50mM Tris/HCL pH=8.0), sonicated, and stored at -80°C until LC-MS/MS analysis. Protein disulfide bridges were reduced with 5mM Tris(2-carboxyethyl) phosphine (TCEP) and alkylated with 10mM 2-Chloroacetamide (CAA) in the dark at 30°C for 30min. For whole proteome analyses, 50μg of proteins was prepared for digestion using the filter-aided sample preparation (FASP) methodology(*10*) and digested with Sequencing Grade Trypsin (1:25; Promega) overnight at 37°C. The samples were then acidified with TFA, and salts were removed using ZipTip C18 pipette tips (Millipore Corp.). The peptides were eluted with 15μl of 60% ACN, 0.1% TFA, dried to completion, and then re-dissolved in 3% ACN, 0.1% formic acid to a final peptide concentration of 0.5μg/μl.

For ADP-ribosylome analyses, 10mg of proteins were diluted 1:12 in PARG buffer (50mM Tris/HCl pH=8, 50mM NaCl, 10mM $MgCl_2$, 250μM DTT) and digested with modified Porcine Trypsin (1:25; Sigma) overnight at 37°C. ADPr-Peptide enrichments were carried out as described (*11, 12*) with the following protocol modifications. Following PARG-mediated PAR-to-MAR peptide ADPr-modification reduction, the

peptides were enriched using Af1521-WT (0.5mL beads/15mg lysate) and eAF1521 (1.0mL beads/15mg lysate) for 2h at 4ºC. The enriched samples were prepared for MS analysis as described previously (*11*).

Liquid chromatography and mass spectrometry analysis

Identification of ADP-ribosylated peptides from untreated and RBN-2397-treated cells was performed on an Orbitrap Lumos mass spectrometer (Thermo Fisher Scientific) coupled to an ACQUITY M class UPLC liquid chromatograph (Waters). We applied an ADPr product-dependent analysis called HCD-PP-EThcD (*13*). Solvent compositions in channels A and B were 0.1% formic acid in the water, and 0.1% formic acid in acetonitrile, respectively. Peptides were loaded onto a nanoEase M/Z Symmetry (Waters) trap column, 18µm × 20 mm, packed with C18 material, 5µm, 100 Å, and separated on an analytical nanoEase M/Z HSS T3 Column (Waters, 75µm × 200 mm) packed with reverse-phase C18 material (Waters, 1.8µm, 100 Å). Peptides were eluted over 110 min at a 300nl/min flow rate. A linear elution gradient protocol was used from 3 to 25% B for 95 min, followed by 35% B for 5 min, and a wash step at 95% B for 5 min, respectively. Full-scan MS spectra (350−2000 m/z) were acquired at a resolution of 120,000, with an AGC target set at $4e^5$ and a maximum injection time of 50ms. High-energy HCD MS/MS spectra (collision energy at 38%) were acquired at a resolution of 30,000, with the AGC target set at $5e^4$ and a maximum injection time of 60ms. Two or more observed ADPr fragment peaks (136.0623, 250.0940, 348.07091, and 428.0372) in the high-energy data dependent HCD scan triggered additional high-quality HCD and EThcD MS/MS scans (resolution of 120,000, AGC target set at $5e^5$, injection time of 240ms, collision energy for HCD scan at 35%).

Qualitative MS data analysis

MS^1 and MS^2 spectra were converted to Mascot generic format (MGF) using Proteome Discoverer, v2.1 (Thermo Fisher Scientific). For the multiple fragmentation techniques (HCD and EThcD) utilized, separate MGF files were created from the raw file for each type of fragmentation. Mascot searches were performed as described (*13*) with the following protocol modifications: the MGFs were searched against the UniProtKB human database (taxonomy 9606, version 20190709), which included 24'905 Swiss-Prot, 34'616 TrEMBL entries, 59'783 decoy hits, and 262 common contaminants. Cysteine carbamido-methylation was set as a fixed modification, and protein N-terminal acetylation and methionine oxidation were set as variable modifications. Finally, S, R, K, D, E, H, C, T, and Y residues were set as variable ADP-ribose acceptor amino acids. The neutral losses of 347.0631 Da and 583.0829 Da from the ADP-ribose were scored in HCD fragment ion spectra (*14*).

Label-free quantification analysis (LFQ)

MS^1-based LFQ was performed using Progenesis QI for Proteomics software (Nonlinear Dynamics) with default settings and the following exceptions. Peptide ions were filtered for charges ranging from +2 to +5. A maximum of the five top-ranked MS/MS spectra per peptide ion were exported with the most intense 200 peaks per spectrum with activated charge-deconvolution and deisotoping options as a Mascot generic formatted file (MGF). MS/MS spectra were searched with Mascot for each type of fragmentation (HCD and EThcD). Mascot searches were carried out as previously described (*15*). A peptide tolerance of 8 ppm and MS/MS tolerance of 0.03 Da were used. Enzyme specificity was set to trypsin, allowing up to four missed cleavages. The MGFs were searched against the target-decoy UniProtKB human database (taxonomy 9606, canonical sequences and reviewed entries only, downloaded on 2022/01/02). N-terminal protein acetylation was set as a variable modification. C, H, S, R, K, D, E, T, and Y residues were set as variable ADPr acceptor sites with a mass shift of 541.0611 Da. Cysteine carbamidomethylation was set as a fixed modification and oxidation as a variable modification on M. The neutral losses from the ADPr 249.0862, 347.0631, and 583.0829 Da were scored in HCD fragment ion spectra. For HCD and EThcD fragment ion spectra, the marker ions at m/z 428.0372, 348.0709, 250.0940, and 136.0623 were ignored for scoring. The Mascot search results were imported into Scaffold and filtered for protein and peptide FDR values of 1%. When multiple precursors were observed for the same peptide, the values were summed up to obtain the total intensity level of the peptide.

Statistical significances were determined using $\log_{10}$-transformed and normalized MS^1 signal intensities of four technical replicates and compared using a two-sided Student's T-test. GO analysis of PARP7 protein targets was performed using Web-Gestalt (Release date 01.04.2019). The online STRING database (version 11.5) was used for the generation of protein interaction networks, and Cytoscape (version 3.9.1) was used for manual annotation and visualization of the STRING networks.

Genetic dependency analysis
PARP7 genetic co-dependencies were determined by comparing gene effect scores (DepMap version 19q3) using the ShineyDepMap tool (version 2). The resulting genetic co-dependency network was visualized in Cytoscape (version 3.9.1). FRA1 and PARP7 expression and CRISPR dependency scores (DepMap version 22Q4) were compared using a linear regression model.

Data Availability
The RNA sequencing data of NCI-H1975 cells generated in this study have been deposited in the Gene Expression Omnibus database under accession code GSE229674. The MS proteomics data generated and analyzed during the current study have been deposited to the ProteomeXchange Consortium via the PRIDE partner repository with the dataset identifier PXD041870. We also used the publicly accessible transcriptomics data set GSE177494 (RBN-2397 treatment of NCI-H1373 cells for 24h). The RNA sequencing data of 1078 cell lines, cell line annotations, and gene dependency scores were downloaded from the portal of the Dependency Map (DepMap) project (https://depmap.org/portal, release: Public 22Q4). Illustrations were created with a licensed version of BioRender.com (License number for Fig. 6G: BY25RAU2YF). All data are available in the main text or the supplementary information.

Statistical Analysis
For all experiments with two groups, p-values were determined by Student's t-test. For all experiments with groups of three or more and one or two independent variables, p-values were determined by one-way or two-way ANOVA with multiple comparison analysis, respectively. Statistical significance is indicated in the figure panels as *p<0.05, **p<0.002, ***p<0.0002 and ****p<0.0001. The number of analyzed biological and/or technical replicates for each experiment is stated in the figure legends.

Supporting Information - Figures

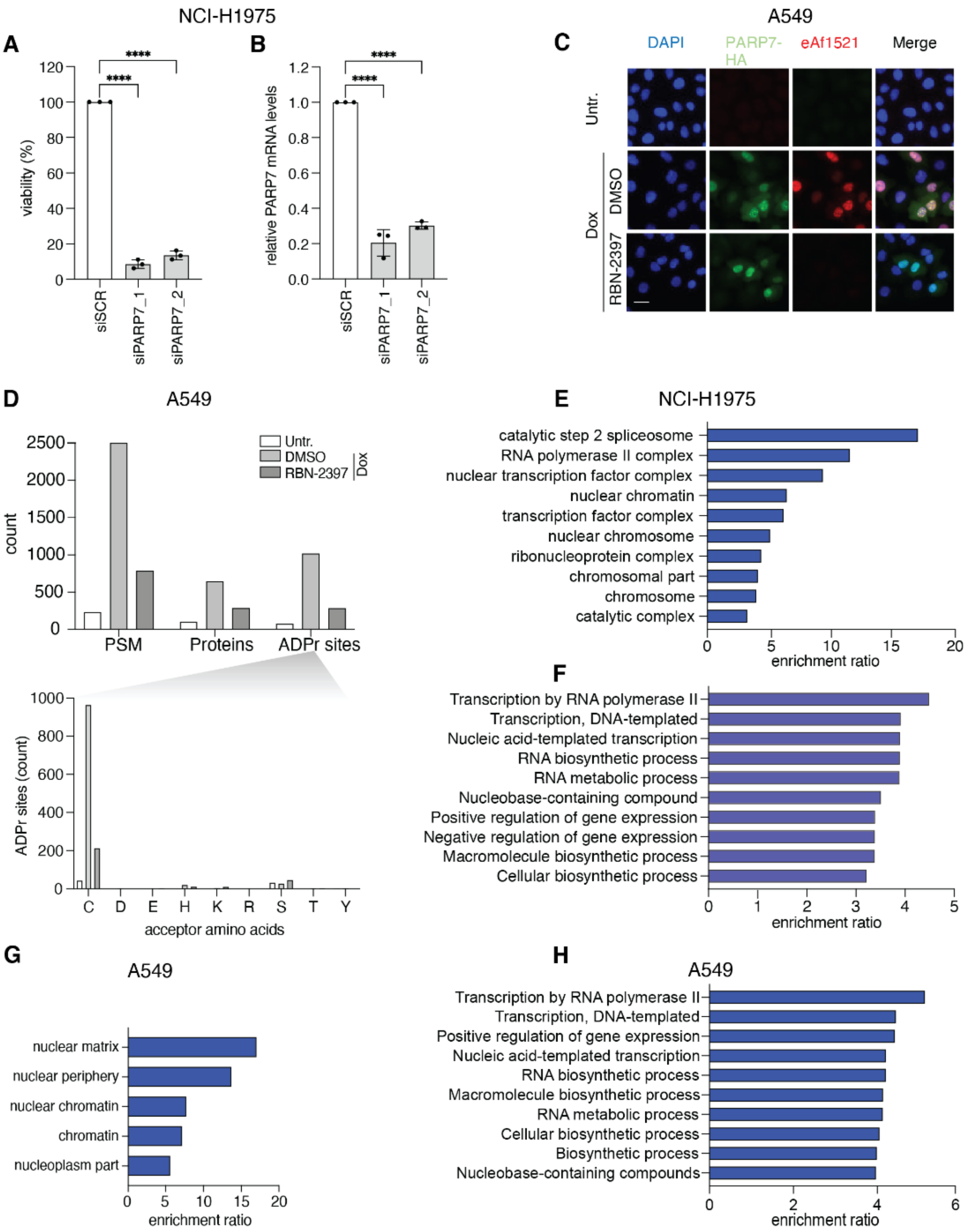

Fig. S1. (A) Cell viability of NCI-H1975 measured by CellTiter-Blue assay following 72h of PARP7 knockdown. Data are depicted as the mean ±SD of N=3 biological replicates. **(B)** RT-qPCR analysis of PARP7 mRNA levels after PARP7 knockdown for 48h. Data are depicted as the mean ±SD of N=3 biological replicates. **(C)** IF analysis of Doxycycline (Dox)-indued HA-tagged PARP7. Cells were treated with 100nM RBN-2397 for 24h in A549 cells or with the solvent control (DMSO) or were left untreated (Untr.). Representative image from a single experiment of N=5 biological replicates. The scale bar represents 20 μm. **(D)** Bar graph of A549 cells treated with Dox alone or in addition with DMSO or RBN-2397 (100nM) for 24h showing the count of unique ADPr-PSMs, unique ADPr-proteins and unique ADPr-sites with ≥95% site-localization confidence (upper). ADP-ribosylated amino acid residue distribution was assessed by EThcD and HCD fragmentation (lower). The experiment was performed once. **(E)** Overrepresentation analysis of proteins exhibiting a decrease in ADP-ribosylation after RBN-2397 (100nM) treatment (from Fig. 1C) in NCI-H1975 cells using GO cellular localization annotations. **(F)** Overrepresentation analysis of proteins exhibiting a decrease in ADP-ribosylation after RBN-2397 treatment (from Fig. 1C) in NCI-H1975 cells using GO annotations. **(G)** Overrepresentation analysis of proteins exhibiting increased ADP-ribosylation after Dox treatment in A549 cells using GO cellular localization annotations. **(H)** Overrepresentation analysis of proteins exhibiting increased ADP-ribosylation after Dox treatment in A549 cells using GO annotations.

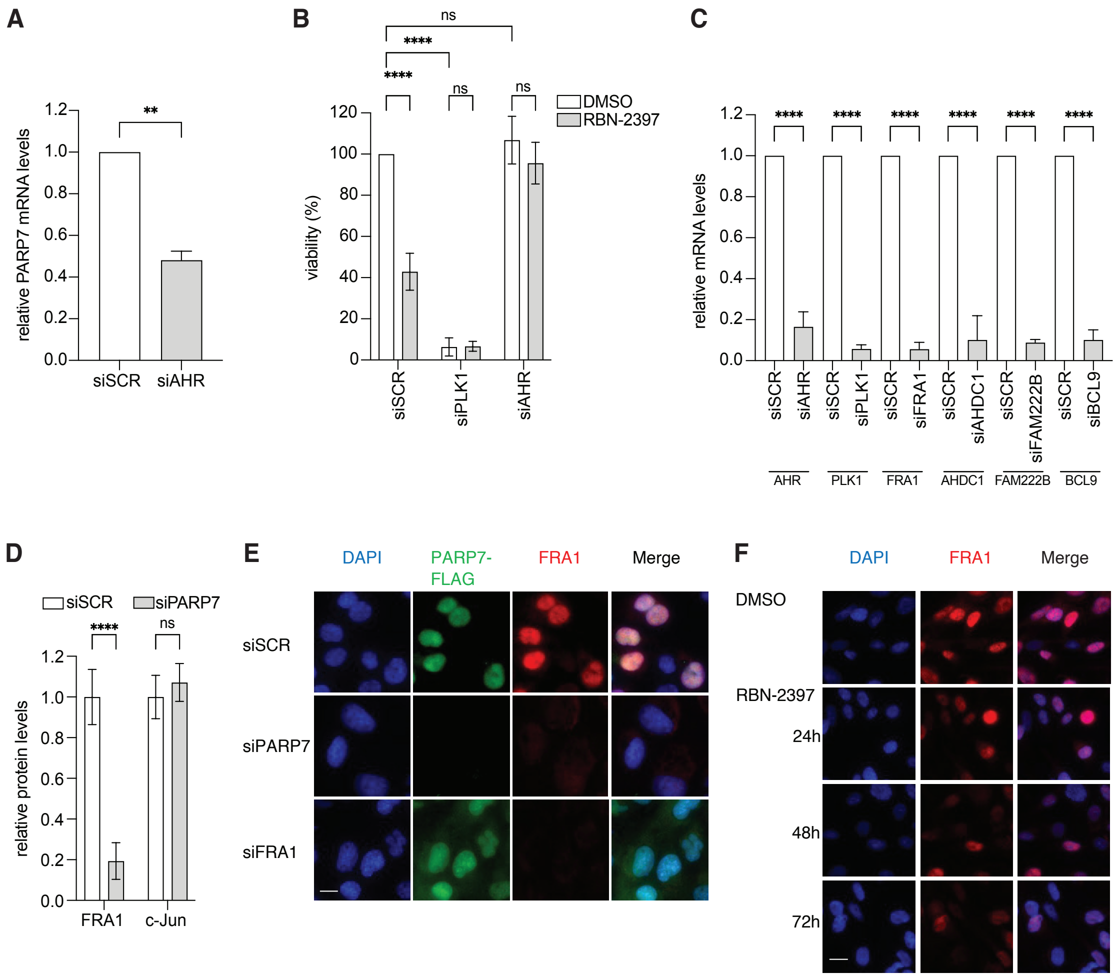

Fig. S2. (A) RT-qPCR analysis of PARP7 mRNA levels following 48h of AHR knockdown. Data are depicted as the mean ±SD of N=3 biological replicates. (**B**) Cell viability of NCI-H1975 cells measured by CellTiter-Blue assay following five days of siPLK1 and siAHR transfection and 100nM RBN-2397 treatment. Data are normalized to the siSCR + DMSO control and shown as the mean ±SD of N=3 biological replicates. **(C)** RT-qPCR analysis of the indicated genes following 48h of knockdown. Data are depicted as the mean ±SD of N=2 biological replicates. (**D**) Quantification of FRA1 and c-JUN immunoblots following the knockdown of PARP7 was performed by normalizing the signal intensities of FRA1 and c-JUN to GAPDH. Data are shown as normalized to siSCR and as the mean ±SD of N=3 biological replicates. **(E)** IF analysis of FRA1 and endogenously FLAG-tagged PARP7 after the knockdown of FRA1 or PARP7 in NCI-H1975 cells. Representative image from a single experiment with N=3 biological replicates. The scale bar represents 20 µm. **(F)** IF analysis of FRA1 after 100nM RBN-2397 treatment for 0, 24, 48, and 72h in NCI-H1975 cells. Representative image from a single experiment of N=3 biological replicates. The scale bar represents 20 µm.

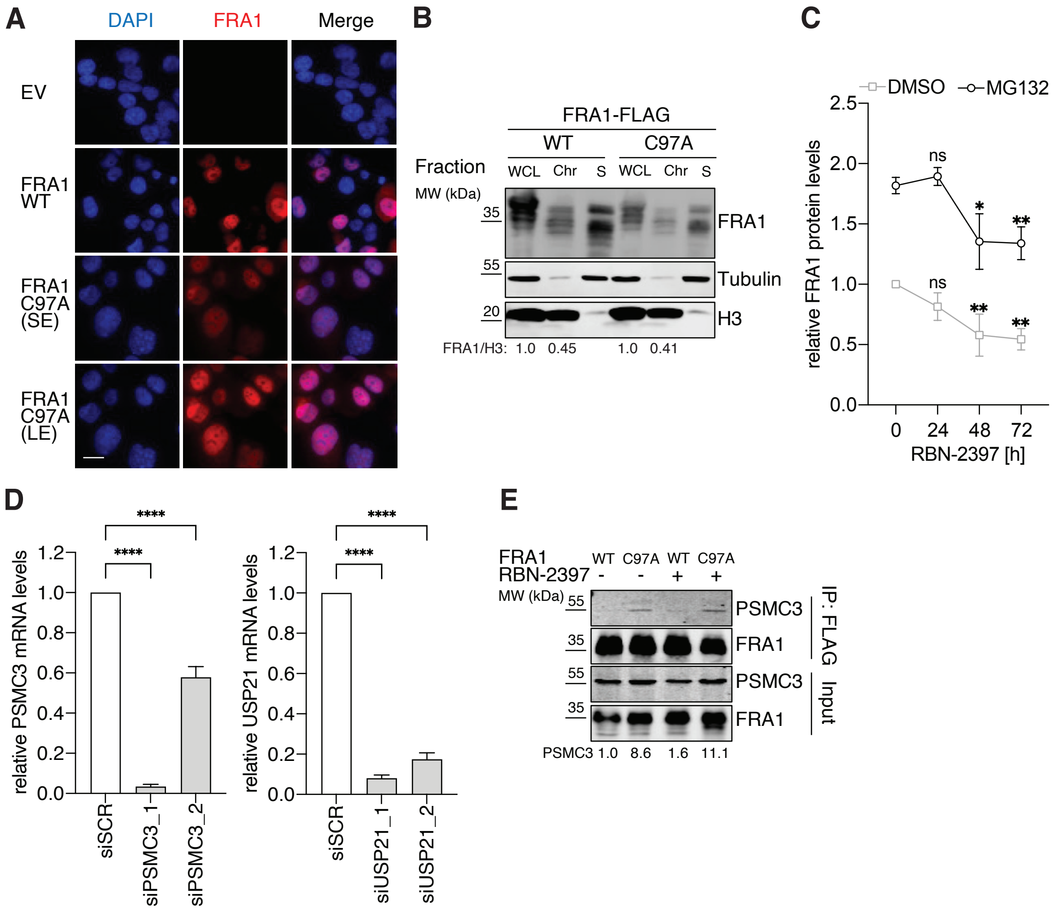

Fig. S3. (A) IF analysis of NCI-H1975 cells overexpressing FRA1 WT or the C97A mutant. Representative image from a single experiment with N=2 biological replicates. The scale bar represents 20 µm. (SE: short exposure; LE: long exposure). **(B)** Immunoblot showing the chromatin fractionation of NCI-H1975 cells ectopically expressing WT or C97A FRA1 (WCL: whole cell lysate; Chr: Chromatin fraction; S: Soluble fraction). Representative image from a single experiment with N=2 biological replicates. FRA1 levels were quantified and normalized to H3 and shown as the mean of N=2. **(C)** Quantification of FRA1 immunoblots was performed by normalizing to GAPDH and shown as the mean ±SD of N=3 biological replicates. **(D)** RT-qPCR analysis of PSMC3 and USP21 following 48h of PSMC3 and USP21 knockdown, respectively. Data are depicted as the mean ±SD of N=2 biological replicates. **(E)** NCI-H1975 cells expressing FRA1-WT or C97A were treated with 100nM RBN-2397 and subjected to IP using anti-FLAG agarose beads. Immunoblotting was performed by probing for PSCMC3 and FRA1. For IP assays, three times more FRA1-C97A expressing cells were used and lysed in the same volume as FRA1-WT expressing cells. Representative image from a single experiment with N=3 biological replicates. Quantification of PSMC3 IP immunoblots was performed by normalizing to the input and is depicted as the mean of N=3.

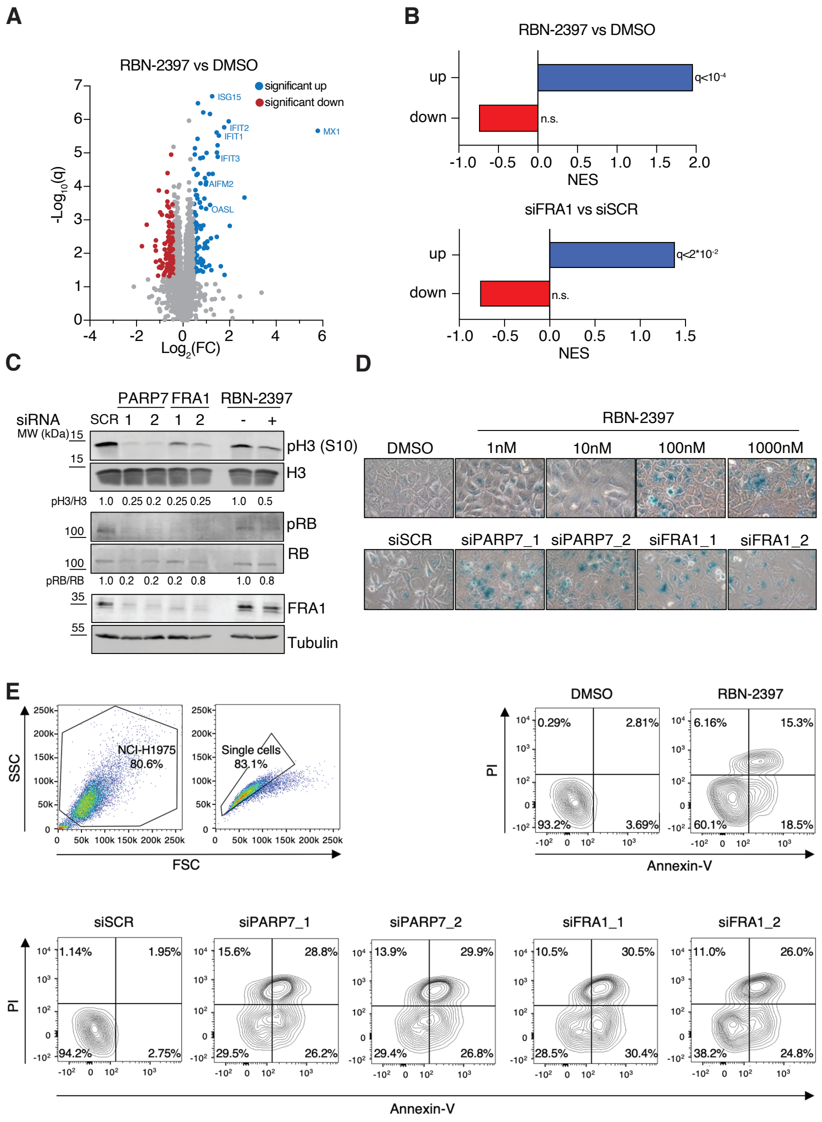

Fig. S4. (A) Volcano plot showing protein expression changes after 24h 100nM RBN-2397 treatment in NCI-H1975 cells. Biological quadruplicates (N=4) for each condition were subjected to whole proteome LC-MS/MS. The FDR <0.05 and a $Log_2(FC) \geq \pm 0.58$ define significant changes in protein expression. **(B)** Overlap between proteome data shown in (A) and the data from Fig. 4A-B, showing up and down-regulated genes, respectively, after siFRA1 and RBN-2397 (100nM) treatment. **(C)** Immunoblot of cell cycle markers after siPARP7, siFRA1, and RBN-2397 (100nM) treatment, respectively, for 48h. Representative image of N=2 biological replicates. Quantification of pH3 and pRB was performed by normalizing signal intensities of pH3 and pRB to H3 and RB, respectively, and is depicted as the mean of N=2. **(D)** β-Galactosidase staining of NCI-H1975 following the knockdown of PARP7 or FRA1 and treatment with increasing concentrations of RBN-2397 for 48h. Representative image from a single experiment with N=3 biological replicates. **(E)** Flow cytometry plots depicting the gating strategy and the single cell distribution using Annexin-V and PI measurements following the indicated treatments. Representative image from a single experiment with N=3 biological replicates.

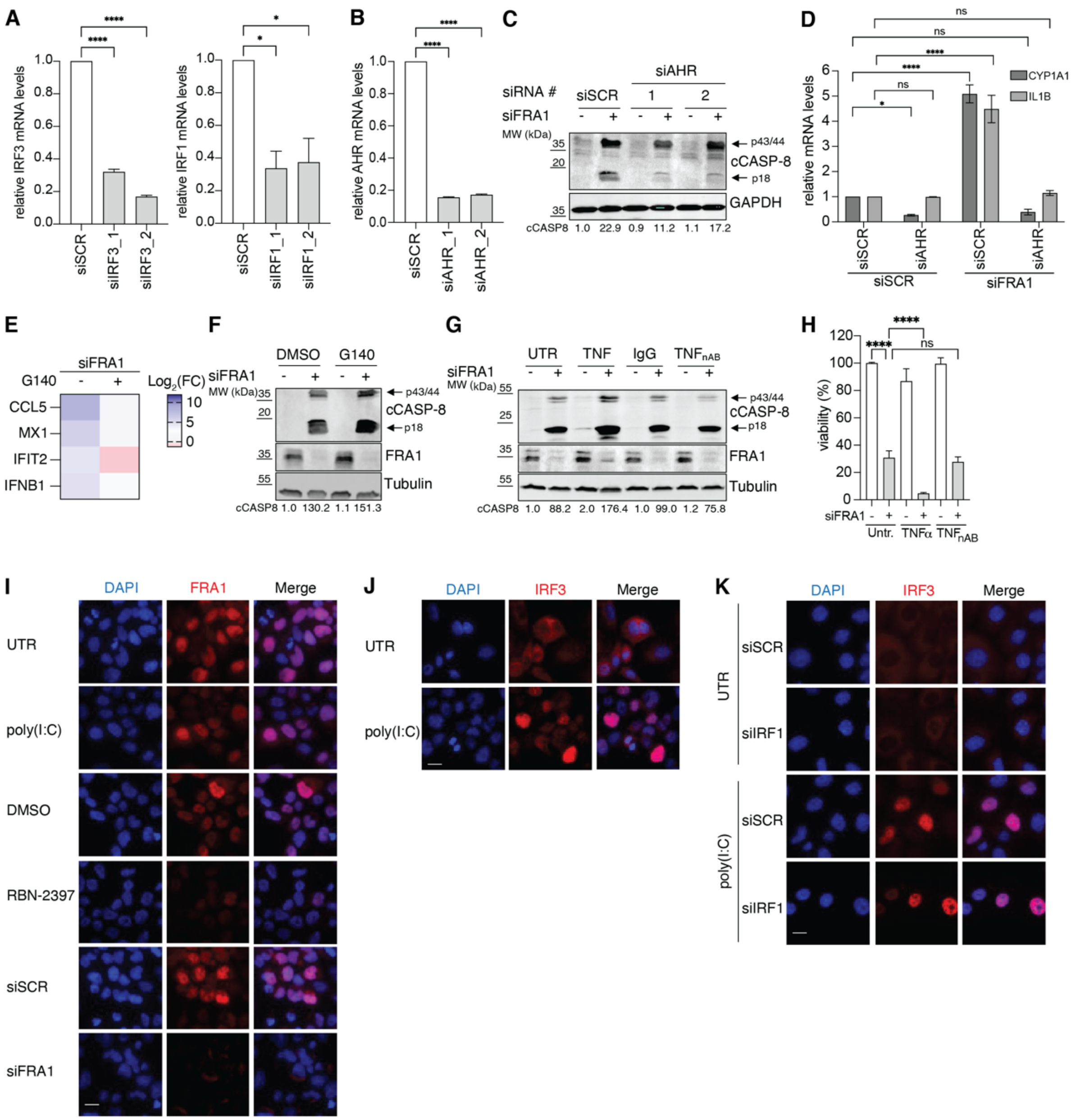
A
relative IRF3 mRNA levels
siSCR
siIRF3_1
siIRF3_2
relative IRF1 mRNA levels
siIRF1_1
siIRF1_2
B
relative AHR mRNA levels
siAHR_1
siAHR_2
C
siRNA #
siSCR
siAHR
siFRA1
MW (kDa)
cCASP-8
p43/44
p18
GAPDH
cCASP8 1.0 22.9 0.9 11.2 1.1 17.2
D
relative mRNA levels
CYP1A1
IL1B
siSCR
siAHR
siFRA1
ns
E
siFRA1
G140
CCL5
MX1
IFIT2
IFNB1
Log2(FC)
F
DMSO
G140
FRA1
Tubulin
cCASP8 1.0 130.2 1.1 151.3
G
UTR
TNF
IgG
TNFnAB
cCASP8 1.0 88.2 2.0 176.4 1.0 99.0 1.2 75.8
H
viability (%)
Untr.
TNFα
I
DAPI
FRA1
Merge
UTR
poly(I:C)
DMSO
RBN-2397
siSCR
siFRA1
J
IRF3
K
siIRF1

Fig. S5. (A) RT-qPCR analysis of IRF3 and IRF1 mRNA levels following 48h of IRF3 and IRF1 knockdown. Data are depicted as the mean ±SD of N=3 biological replicates. **(B)** RT-qPCR analysis of AHR mRNA levels following 48h of AHR knockdown. Data are depicted as the mean ±SD of N=2 biological replicates. **(C)** Immunoblot of NCI-H1975 cells after the co-treatment with siFRA1 and siAHR for 48h. Representative image from a single experiment with N=3 biological replicates. Quantification of cleaved CASP8 immunoblots was performed by normalizing to GAPDH and indicated as the mean of N=3. **(D)** RT-qPCR analysis of *CYP1A1* and *IL1B* mRNA levels following single or double knockdown of FRA1 and AHR. Data are depicted as the mean ±SD of N=2 biological replicates. **(E)** Heat map showing RT-qPCR analysis of NCI-H1975 cells after G140 treatment (10μM). Data is normalized to siSCR + DMSO and shown as the mean of N=2 biological replicates. **(F)** Immunoblot of NCI-H1975 cells after the co-treatment with siFRA1 and G140 (10μM) for 48h. Representative image from a single experiment with N=3 biological replicates. Quantification of cleaved CASP8 immunoblots was performed by normalizing to tubulin and indicated as the mean of N=3. **(G)** Immunoblot of NCI-H1975 cells after the co-treatment with siFRA1, TNF (100ng/ml), and a TNF neutralizing antibody (1μg/ml) for 72h. Representative image from a single experiment with N=3 biological replicates. Quantification of cleaved CASP8 immunoblots was performed by normalizing to tubulin and indicated as the mean of N=3. **(H)** Cell viability of NCI-H1975 cells was measured by CellTiter-Blue assay following 72h of co-treatment with siFRA1, TNF (100ng/ml), and a TNF neutralizing antibody (1μg/ml). Data are normalized as indicated and shown as the mean ±SD of N=3 biological replicates. **(I)** IF analysis of FRA1 after poly(I:C) transfection (10ng/ml, 3h), FRA1 knockdown (48h) and PARP7 inhibition (48h) in NCI-H1975 cells. Representative image from a single experiment with N=3 biological replicates. The scale bar represents 20 μm. **(J)** IF analysis of IRF3 after poly(I:C) transfection (10ng/ml) for 3h in NCI-H1975 cells. Representative image from a single experiment with N=3 biological replicates. The scale bar represents 20 μm. **(K)** IF analysis of IRF3 after the knockdown of IRF1 for 48h and poly(I:C) treatment (10ng/ml) for 3h in NCI-H1975 cells. Representative image from a single experiment with N=3 biological replicates. The scale bar represents 20 μm.

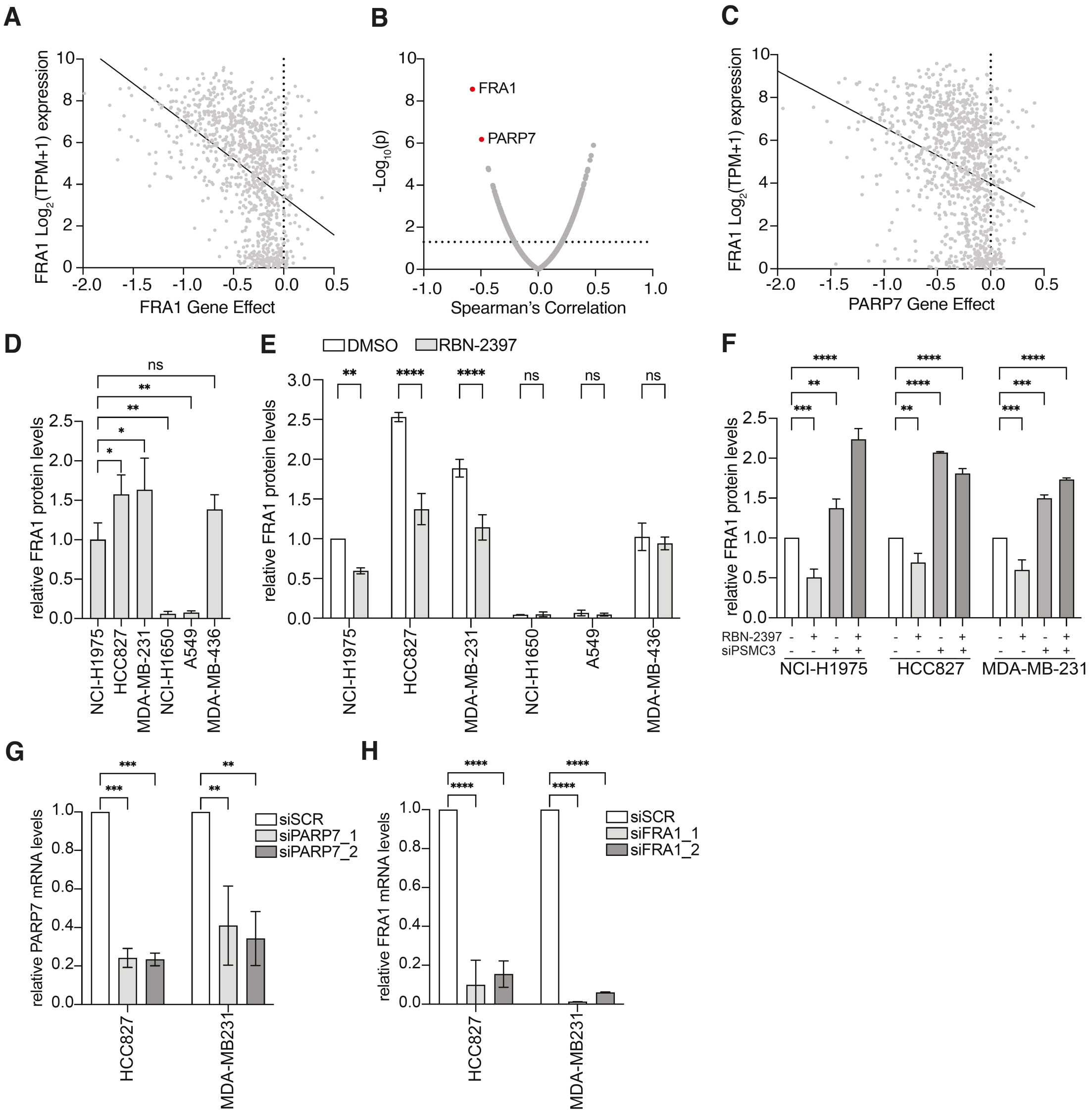
A
FRA1 Log2(TPM+1) expression
FRA1 Gene Effect
B
FRA1
PARP7
-Log10(p)
Spearman's Correlation
C
FRA1 Log2(TPM+1) expression
PARP7 Gene Effect
D
relative FRA1 protein levels
NCI-H1975
HCC827
MDA-MB-231
NCI-H1650
A549
MDA-MB-436
E
DMSO
RBN-2397
relative FRA1 protein levels
F
relative FRA1 protein levels
RBN-2397
siPSMC3
NCI-H1975
HCC827
MDA-MB-231
G
relative PARP7 mRNA levels
siSCR
siPARP7_1
siPARP7_2
HCC827
MDA-MB231
H
relative FRA1 mRNA levels
siSCR
siFRA1_1
siFRA1_2
HCC827
MDA-MB231

Fig. S6. (A) Scatter plot showing the linear relationship between FRA1 mRNA levels and FRA1 genetic dependency. **(B)** Volcano plot showing the significance of the correlation between FRA1 mRNA levels and the genetic dependency of all assessed genes. **(C)** Scatter plot showing the linear relationship between FRA1 mRNA levels and PARP7 genetic dependency. **(D)** Quantification of FRA1 immunoblots was performed by normalizing signal intensities of FRA1 to tubulin. The mean ±SD of N=3 biological replicates were compared to NCI-H1975 cells. **(E)** Quantification of FRA1 immunoblots was performed by normalizing signal intensities of FRA1 to tubulin. Expression levels (mean ±SD of N=3 biological replicates) were compared to the DMSO control of NCI-H1975 cells. **(F)** Quantification of FRA1 immunoblot was performed by normalizing signal intensities of FRA1 to tubulin. Expression levels (mean ±SD of N=3 biological replicates) were compared to the siSCR + DMSO control of each cell line. **(G)** RT-qPCR analysis of PARP7 mRNA levels following 48h of PARP7 knockdown in HCC827 and MDA-MB-231 cells, respectively. Data are depicted as the mean ±SD of N=2 biological replicates. **(H)** RT-qPCR analysis of FRA1 mRNA levels following 48h of FRA1 knockdown in HCC827 and MDA-MB-231 cells, respectively. Data are depicted as the mean ±SD of N=2 biological replicates.

Dataset S1. (Separate excel file)
Differentially ADP-ribosylated peptides after RBN-2397 treatment in NCI-H1975 cells.

Dataset S2. (Separate excel file)
Differentially ADP-ribosylated proteins after PARP7 overexpression in A549 cells treated with or without RBN-2397.

Dataset S3. (Separate excel file)
Differentially expressed genes from RNA sequencing analysis of NCI-H1975 cells treated with siFRA1.

Dataset S4. (Separate excel file)
Differentially expressed genes from RNA sequencing analysis of NCI-H1975 cells treated with RBN-2397.

Dataset S5. (Separate excel file)
Differentially expressed proteins from LC-MS/MS analysis of NCI-H1975 cells treated with RBN-2397.

Dataset S6. (Separate excel file)
Oligonucleotide sequences used in this study.

Supporting Information - References

1. J. L. Schmid-Burgk, K. Höning, T. S. Ebert, V. Hornung, CRISPaint allows modular base-specific gene tagging using a ligase-4-dependent mechanism. *Nature Communications* **7**, 12338 (2016).
2. C. Swanton *et al.*, Regulators of Mitotic Arrest and Ceramide Metabolism Are Determinants of Sensitivity to Paclitaxel and Other Chemotherapeutic Drugs. *Cancer Cell* **11**, 498-512 (2007).
3. G. Grimaldi *et al.*, PKD-dependent PARP12-catalyzed mono-ADP-ribosylation of Golgin-97 is required for E-cadherin transport from Golgi to plasma membrane. *Proceedings of the National Academy of Sciences* **119**, e2026494119 (2022).
4. M. Hatakeyama *et al.*, SUSHI: an exquisite recipe for fully documented, reproducible and reusable NGS data analysis. *BMC Bioinformatics* **17**, 228 (2016).
5. A. Dobin *et al.*, STAR: ultrafast universal RNA-seq aligner. *Bioinformatics* **29**, 15-21 (2013).
6. A. Frankish *et al.*, GENCODE 2021. *Nucleic Acids Res* **49**, D916-d923 (2021).
7. Y. Liao, G. K. Smyth, W. Shi, The Subread aligner: fast, accurate and scalable read mapping by seed-and-vote. *Nucleic Acids Res* **41**, e108 (2013).
8. M. I. Love, W. Huber, S. Anders, Moderated estimation of fold change and dispersion for RNA-seq data with DESeq2. *Genome Biology* **15**, 550 (2014).
9. G. Yu, L. G. Wang, Y. Han, Q. Y. He, clusterProfiler: an R package for comparing biological themes among gene clusters. *Omics* **16**, 284-287 (2012).
10. J. R. Wiśniewski, A. Zougman, N. Nagaraj, M. Mann, Universal sample preparation method for proteome analysis. *Nature Methods* **6**, 359-362 (2009).
11. K. Nowak *et al.*, Engineering Af1521 improves ADP-ribose binding and identification of ADP-ribosylated proteins. *Nature Communications* **11**, 5199 (2020).
12. R. Martello *et al.*, Proteome-wide identification of the endogenous ADP-ribosylome of mammalian cells and tissue. *Nature Communications* **7**, 12917 (2016).
13. V. Bilan, M. Leutert, P. Nanni, C. Panse, M. O. Hottiger, Combining Higher-Energy Collision Dissociation and Electron-Transfer/Higher-Energy Collision Dissociation Fragmentation in a Product-Dependent Manner Confidently Assigns Proteomewide ADP-Ribose Acceptor Sites. *Analytical Chemistry* **89**, 1523-1530 (2017).
14. P. M. Gehrig *et al.*, Gas-Phase Fragmentation of ADP-Ribosylated Peptides: Arginine-Specific Side-Chain Losses and Their Implication in Database Searches. *J Am Soc Mass Spectrom* **32**, 157-168 (2021).
15. M. Leutert *et al.*, Proteomic Characterization of the Heart and Skeletal Muscle Reveals Widespread Arginine ADP-Ribosylation by the ARTC1 Ectoenzyme. *Cell Rep* **24**, 1916-1929.e1915 (2018).
